# The Acidic Exosomal miR-1246/WASF3 Axis Regulates Hepatic Stellate Cell Activation and Stiff ECM Remodeling to Promote Pancreatic Ductal Adenocarcinoma Liver Metastasis

**DOI:** 10.7150/ijbs.132772

**Published:** 2026-08-21

**Authors:** Dongqi Li, Xiangyu Chu, Fusheng Zhang, Yongsu Ma, Weikang Liu, Ping Li, Xiaocui Fang, Chen Wang, Xiaodong Tian, Yanlian Yang, Yinmo Yang

**Affiliations:** 1 Department of Hepatobiliary ad Pancreatic Surgery, Peking University First Hospital, Beijing 100034, China; 2 CAS Key Laboratory of Standardization and Measurement for Nanotechnology, CAS Key Laboratory of Biological Effects of Nanomaterials and Nanosafety, National Center for Nanoscience and Technology, Beijing, China; 3 Department of General Surgery, Beijing Friendship Hospital, Capital Medical University, State Key Lab of Digestive Health, National Clinical Research Center for Digestive Diseases, Beijing, 100050, China; 4 University of Chinese Academy of Sciences, Beijing 100049, China

**Keywords:** Pancreatic ductal adenocarcinoma, Exosomal miRNA, Stiff extracellular matrix, pre-metastatic niche, Liver metastasis

## Abstract

Liver metastasis is a major factor contributing to the poor prognosis of pancreatic ductal adenocarcinoma (PDAC). The formation of pre-metastatic niche (PMN) initiates the process of liver metastasis. Exosomes (Exos) act as key mediators of crosstalk between the tumor microenvironment (TME) and the PMN to activate hepatic stellate cells (HSCs) and remodel the stiff extracellular matrix (ECM). In this study, we isolated Exos derived from PDAC cells cultured under acidic conditions and demonstrated that these Exos significantly activate HSCs and promote the remodeling of the stiff ECM, thereby promoting the stemness, migration, and invasion of PDAC cells. High expression of exosomal miR-1246 was screened by miRNA-sequencing, and Wiskott-Aldrich syndrome protein Family Member 3 (WASF3) was identified as the target of miR-1246. Mechanistically, exosomal miR-1246 activates HSCs to remodel the ECM by targeting WASF3 and stimulating the phosphatidylinositol 3-kinase-serine/threonine protein kinase (PI3K/Akt) pathway. Notably, RNA-binding protein immunoprecipitation (RIP) and miRNA pull-down assays were performed to identify that Human Antigen R (HuR) contributes to the enrichment of miR-1246 into Exos. Collectively, exosomal miR-1246 activates HSCs and remodels the stiff ECM to promote liver metastasis, and it may serve as a potential diagnostic and prognostic marker for PDAC liver metastasis.

## Introduction

Pancreatic ductal adenocarcinoma (PDAC) is one of the most aggressive malignancies, with a 5-year relative survival rate of 13% 1. Its poor prognosis is largely due to late detection and a high propensity for metastasis 2. The liver is the most common site of metastasis, occurring in approximately 80% of patients with PDAC, who thereby lose the opportunity for radical surgery 3, 4. The complex interplay between PDAC cells and the hepatic microenvironment contributes to resistance to conventional chemoradiotherapy, further worsening patient outcomes 5. Therefore, elucidating the mechanisms underlying PDAC liver metastasis and identifying early diagnostic and therapeutic biomarkers are crucial for improving prognosis.

Exosomes (Exos) are small extracellular vesicles (EVs) of endocytic origin, typically 30-150 nm in diameter, containing non-coding RNAs, DNAs, proteins, metabolites, and lipids 6. Notably, severe acidosis caused by excessive metabolic acid production and poor perfusion promotes Exos release from PDAC cells and enhances their aggressiveness 7, 8. Exos mediate intercellular communication and play key roles in regulating PDAC progression 9, 10. Organ-specific tumor metastasis begins with the formation of a pre-metastatic niche (PMN), composed of various cell types and complex intercellular interactions that facilitate the colonization and growth of circulating tumor cells (CTCs) 11. Tumor-derived exosomes (TDEs) promote PMN formation by inducing angiogenesis, establishing an immunosuppressive microenvironment, remodeling the extracellular matrix (ECM), and reprogramming metabolism 12-14. Hepatic stellate cells (HSCs), which are multifunctional liver-resident cells, can transform into myofibroblast-like cells upon stimulation, driving ECM remodeling through the secretion of collagen and fibronectin (FN) 15. The remodeled ECM would recruit immunosuppressive cells and induce abnormal signaling pathways in tumor cells by altering tissue stiffness and collagen fiber alignment, thereby fostering a metastasis-promoting microenvironment 16. Investigating how TDEs activate HSCs is essential to elucidate the role of Exos in mediating communication between primary tumors and the hepatic PMN.

As key regulators of gene expression, TDE miRNAs mediate intercellular communication and influence tumor progression. For example, exosomal miR-301a promotes M2 macrophage polarization via the phosphatase and tensin homolog/phosphatidylinositol 3-kinase subunit gamma (PTEN/PI3Kγ) pathway, facilitating PDAC metastasis 17. miR-1246 is an oncogenic miRNA in PDAC, enhancing cancer stem cell-like properties and gemcitabine (GEM) resistance by targeting Cyclin G2 (CCNG2) 18. Profiling of plasma Exos from both PDAC xenograft mice and patients consistently shows significant TDE miR-1246 overexpression 19. Notably, TDE miR-1246 activates the ERK and serine/threonine protein kinase (Akt) pathways and upregulates α-smooth muscle actin (α-SMA) and fibrosis-related genes in pancreatic stellate cells (PSCs), indicating its role in mediating crosstalk between PDAC and stromal cells 20. Therefore, we hypothesize that TDE miR-1246 activates HSCs, inducing pro-fibrotic protein secretion and ECM remodeling, thereby shaping the hepatic PMN to promote PDAC liver metastasis.

The Wiskott-Aldrich syndrome protein family member 3 (WASF3) belongs to the WAVE family and functions downstream of Ras-related C3 botulinum toxin substrate 1 (Rac1) to mediate Rac-induced actin polymerization during lamellipodium formation 21, 22. WASF3 participates in essential cellular processes, including cytokinesis, motility, and adhesion, and regulates the expression of key molecules like matrix metalloproteinases (MMPs), mitogen-activated protein kinase (MAPK), and Snail 23, 24. Although public database analyses suggest that WASF3 may act as a tumor suppressor in PDAC, its role in stromal cells, especially in HSC activation, remains poorly characterized. In this study, we identified WASF3 as a target of exosomal miR-1246. Through WASF3 overexpression experiments, we demonstrated that miR-1246 activates HSCs and remodels the ECM by suppressing WASF3, thereby forming a stiff microenvironment.

In this study, exosomes derived from the acidic culture supernatant (Exo-A) were isolated and shown to activate HSCs, thereby promoting collagen I and FN secretion and increasing ECM stiffness. Notably, the stiffened ECM directly enhanced the stemness of PDAC cells, providing a mechanistic basis for liver metastasis. Subsequent miRNA sequencing revealed significant miR-1246 enrichment in Exo-A. Using an “Exos education” mouse model, we demonstrate that miRNA-1246 is crucial in driving HSC activation and liver metastasis *in vivo*. Mechanistically, exosomal miR-1246 downregulated WASF3 expression and activated HSCs through the PI3K-Akt pathway, thereby facilitating PDAC liver metastasis. Therefore, TDE miR-1246 mediates crosstalk between tumor cells and the hepatic PMN and may be a potential biomarker and therapeutic target for PDAC liver metastasis.

## Materials and Methods

### Cell culture

The human PDAC cell lines-BxPC-3, AsPC-1, PANC-1, T3M4, Patu-8988, and the normal pancreatic ductal epithelium: hTERT-HPNE, were obtained from the American Type Culture Collection (ATCC, USA); AsPC-1, PANC-1, T3M4 Patu-8988, hTERT-HPNE and 293T were cultured with Dulbecco's modified Eagle's medium (DMEM, Gibco, USA), BxPC-3 was cultured in RPMI-1640 medium (Gibco, USA). All culture media contained 10% fetal bovine serum (FBS, Gibco, USA), and 1% penicillin-streptomycin.

### Exos isolation and analyzation

Exos were isolated from 500 ml cell culture supernatant. First, differential centrifugation (1,500g, 4 °C, 5 min; 2,500g, 4 °C, 20-30 min) was performed to remove cell debris and particles. The supernatant was then filtered through a 0.22 µm filter and finally purified exosome precipitates were obtained by centrifugation in an ultracentrifuge for 90 min (100,000g, 4 °C) 25.

Exos were analyzed for size and concentration using a nanoparticle tracking assay (NTA, NanoSight NS3000, UK). Exosome morphology was examined by transmission electron microscopy (TEM, Hitachi H-7700, German). Exosome biomarkers (CD9, heat shock protein 70 (HSP70), tumor susceptibility gene 101 (TSG101)) were used to identify exos and calnexin was used as negative control for parental cells.

### Exos uptake assay

Exos and PKH67 dye (Sigma, USA) were incubated at room temperature for 5min, and ended the reaction by adding 0.3% BSA. Then enriched the PKH67-labeled exos by ultracentrifugation for 70 min. The labeled exos were co-incubated with pancreatic cancer cells for 5h, and the nuclei were stained with DAPI (Invitrogen, USA) for 10min. Finally, captured representative photographs by confocal microscopy (Leica, Germany) 26, 27.

### Western blotting (WB)

Washed cells twice with PBS and extracted proteins by Pierce IP lysis buffer (ThermoFisher Scientific, USA) containing protease inhibitors (87785, ThermoFisher Scientific, USA) and phosphatase inhibitors (A32957, ThermoFisher Scientific, USA). After measuring protein concentration, loaded these samples onto a 10% Tris-HCl gradient gel (Invitrogen, USA) before being transferred to the PVDF membranes. Then, blocked the membranes with 5% skimmed milk for 2 hours, and incubated the membranes with primary antibodies, including Calnexin (10427-2-AP, Proteintech), HSP70 (ab181606, Abcam), TSG101 (ab125011, Abcam), CD9 (ab307085, Abcam), GAPDH (60004-1-Ig, Proteintech), Alpha smooth muscle actin (α-SMA, 14395-1-AP, Proteintech), Fibronectin (15613-1-AP, Proteintech), Collagen I (14695-1-AP, Proteintech), WASF3 (17932-1-AP, Proteintech), CD44 (15675-1-AP, Proteintech), ALDH1A (YT5157, Immunoway), OCT4 (11263-1-AP, Proteintech), p-PI3K (YP0765, Immunoway), PI3K (60225-1-Ig, Proteintech), Akt (9272S, Cell Signaling Technology), p-Akt (9271T, Cell Signaling Technology).

### Quantitative reverse transcriptase PCR (qRT-PCR)

Separated total RNA from exos using miRNeasy Mini Kit (QIAGEN, Germany), and isolated cellular RNA through Trizol reagent (Invitrogen, USA). Then, synthesized first-strand cDNA from 2μg total RNA by ReverseTra Ace qPCR RT kit (FSQ101/201, TOYOBO, Japan). Conducted qRT-PCR by adding SYBR Green Realtime PCR Master Mix (Q711-02, Vazyme Biotech, China). Lastly, determined relative expression levels of target miRNAs and genes by using the 2^^-△△CT^ method. Primers for RT-qPCR were as follows:

### RBP immunoprecipitation (RIP) assay

RIP assay was conducted using Magna Nuclear RIP™ kit (Millipore). According to the manufacturer's protocol, cell lysates (3 × 10⁷ cells) were immunoprecipitated with magnetic beads coupled to an anti-HuR antibody (11910-1-AP, Proteintech). The retrieved RNA was then analyzed by qRT-PCR, and fold enrichment was calculated using the 2^^-ΔΔCT^ method.

### Dual Luciferase Report Assay

The WASF3 3'-UTR (wild/mutant) vectors were constructed by Syngenbio (Qingdao, China). Then, transfected LX-2 cells with miR-1246 and the constructed vectors using Lipofectamine 3000. After 48h, dual luciferase reporter kit (Beyotime Biotechnology, China) was used to measure the relative intensities of fluorescence 28.

### MiRNAs and plasmids transfection

MiRNA-1246 mimics, inhibitors, and corresponding negative control miRNAs were purchased from Ribio (Guangzhou, Guangdong, China). Transfected these miRNAs (5nM) into BxPC-3 cells using Lipofectamine RNAiMAX reagent (ThermoFisher Scientific, USA). Changed the medium after 6-8 hours' transfection, and harvested cells for subsequent experiments after incubation 48 hours.

The WASF3 negative control (NC) plasmids and overexpression (OE) plasmids, the HuR NC plasmids and shRNA plasmids were obtained from Syngenbio (Qingdao, China). Transfected BxPC-3 cells with NC/OE plasmids using Lipofectamine 3000 (ThermoFisher Scientific, USA). After 48 hours, qRT-PCR and protein gel electrophoresis were performed to detect the transfection efficiency of WASF3.

### Oil red O staining

Firstly, fixed the LX-2 cells 20-30 min, washed them twice with MQ water, and added 2ml 60% isopropanol solution for 20-30s. Next, stained the LX-2 cells with freshly prepared oil red staining solution (liquid A: liquid B = 3:2, prepared 10min in advance), for 10-20 min. Then, washed the LX-2 cells with 60% isopropanol solution for 20-30s, and with MQ water for five times. Further, the nuclei were stained with Mayer hematoxylin stain for 1-2 min. Finally, added 2 ml Oil Red Buffer and incubated for 1min, followed by 2 ml MQ water for observation 29.

### Flow Cytometry

Firstly, cells were digested, fixed with 4% paraformaldehyde (PFA), perforated the membrane, and blocked with 5% BSA. Then, incubated cells with anti-CD133, WASF3 antibodies and used Alexa Fluor 488 (ThermoFisher Scientific, USA) to conjugate primary antibodies. Finally, assessed the expression of CD133/WASF3 of 10,000 cells by flow cytometry.

### Cell Counting Kit-8 (CCK8)

Detected cellular proliferative capacity by Cell Counting Kit-8 (CCK-8, Dojindo, Japan). Specifically, seeded LX-2 and BxPC-3 cells (1×10^4^ cells/well) in 96-well plates, and incubated for 24, 48 and 72 hours. Then, diluted CCK-8 solution to 10%, added 100 μL to the 96-well plate and incubated for 2 h. Finally, measured the absorbance of 450 nm and 630 nm by using chemiluminescence instrument (Molecular Devices, SpectraMax i3).

### Cell colony formation

Seeded 1×10^3^ LX-2/BxPC-3 cells in six-well plates and replaced fresh medium every 2-3 days. After 14 days incubation, discarded the medium and washed them with PBS. Then, fixed these colonies with 4% PFA for 20 min, stained with 0.1% crystal violet, and washed three times with PBS. Colonies were quantified using ImageJ software.

### Cell migration/invasion

The transwell model (8μm pore size, Corning, USA) was constructed to assess cell migration/invasion. Specifically, FBS-free cell suspension (200 μL) was seeded in the upper chamber (pretreated with Matrigel matrix (Corning Incorporated, USA) for invasion assay), and added 600μL FBS medium containing 10% (20% for invasion) to the lower chamber. After incubating 48h, fixed the membranes with 4% PFA, stained with 0.1% crystal violet, washed twice with PBS, and took images under a microscope.

### Tumor stem cell spheroid formation

Firstly, inoculate 1000 BxPC-3 cells/well in six-well low adsorption plates, and add 2ml stem cell culture medium (containing: serum-free DMEM/F12 medium: B27 = 50:1, 20ng/ml of EGF (Beyotime Biotechnology, China) and 100 ng/ml of bFGF (Beyotime Biotechnology, China)) to each well. Then, added and discarded 1ml fresh stem cell medium daily. After 14 days of incubation, observe pancreatic cancer stem cell spheres under the microscope.

### Immunofluorescence (IF)

Fixed cells with 4% PFA, washed with PBS, and blocked with 5% BSA. Next, added primary antibodies for overnight incubation at 4 ℃, and secondary antibodies- Alexa Fluor 488 (ThermoFisher Scientific, USA) was used to conjugate primary antibodies. Then, stained the nucleus with DAPI (Invitrogen, USA), and observed images under confocal microscope.

### ECM extraction

Added 0.2% gelatin solution to a 6-well plate and incubated at 37 °C for 1h, then aspirated the solution and washed with PBS for 3min. Next, added 1% glutaraldehyde solution and incubated at room temperature for 30 min, followed by adding 1 M ethanolamine solution and incubated for another 30 min, then washed with PBS.

Next, incubated LX-2 cells on above plates until the density was on 80-90%, added decellularization solution and treated the plates at 4 °C for 48 h. Finally, added 2 ml 0.5% sodium deoxycholate and incubated 1 h at room temperature, followed by adding 2 ml DNAase (100 ng/ml) and incubated at 37 °C for 1 h.

### Atomic force microscopy (AFM)

Fresh liver tissue was harvested and embedded in Tissue Tek Optimum. Frozen sections of 20 μm thickness were prepared using a cryostat. After thawing, the sections were washed three times with PBS to remove residual embedding compound. Using a microsphere-modified probe (SAA-SPH-5UM, Bruker, Germany) with a 10 μm diameter microsphere, AFM were performed at room temperature in PBS. The parameter settings were as follows: Peak Force Quantitative Nanomechanical Mapping (QNM) mode was selected; the probe spring constant was set to 0.5 N/m; a 20 × 20 μm grid was scanned with an amplitude of 15 μm; and the peak force setpoint frequency was 5 kHz 30. The elastic modulus of the liver tissue was determined by randomly measuring ten points.

### Immunohistochemistry (IHC)

Firstly, fixed tissue sections with 4% PFA for 48h, deparaffinized them in xylene, rehydrated them by ethanol, and washed them by PBS. Next, blocked endogenous peroxidase activity. Specifically, heated tissue sections in 0.01 M citrate buffer for 10-15 min, then cooled them for 10-20 min, and blocked them with 10% goat serum for 60 minutes. Then, incubated sections overnight at 4 ℃ with primary antibodies, and secondary antibodies was used to conjugate primary antibodies, then stained them with diaminobenzidine (DAB). Finally, Mayer hematoxylin (Biodee, China) was performed to stain nuclei, and captured five random images per section.

### Exos “education” assay

All following protocols approved by the Ethics Committee of the National Centre for Nanoscience and Technology. Specifically, ordered six-week-old nude mice from Charles River Co., Ltd. (Beijing). Then, the experimental groups were injected with 100 μL BxPC-3 cell-derived exos (concentration: 200 μg/ml) via tail vein, and the control group was injected with 100 μL PBS, all mice were injected for 21 days. After exosome "education", the livers of the experimental/control groups were subjected to IHC.

### *In situ* xenograft nude mouse model

After exos “education”, mice were subjected to in-situ xenograft models. Firstly, sterilized and incised the left upper abdominal skin of the mice to expose the pancreas, then injected 50 µL BxPC-3 cells (5×10^6^ cells/mL). Executed these mice after 6-7 weeks' inoculation, then observed and counted the number of liver metastasis of PDAC in each group.

### Subcutaneous graft nude mouse model

Six-week-old nude mice were provided by Charles River Co., Ltd. All performed procedures were according to the protocol approved by the Ethics Committee of the National Center for Nanoscience and Technology. Briefly, inoculated 100µL BxPC-3 cells (1×10^7^ cells/mL) into the bilateral axilla of mice. After 2 months inoculation or when the maximum diameter reached 20mm, the mice were executed. Finally, observed the tumor sizes of the control and experimental groups, and sent them for IHC.

### Statistical analysis

All the acquired data were from three independent experiments and analyzed by GraphPad Prism 9.5.0 software. Mean ± standard deviation (SD) was shown for all results, and P < 0.05 represented statistical significance (∗p < 0.05, ∗∗p < 0.01, ∗∗∗p < 0.001, ∗∗∗∗p < 0.0001).

## Results

### Exo-A promotes PDAC liver metastasis by activating HSCs and establishing a pro-fibrotic niche

The acidic tumor microenvironment (TME) of PDAC promotes tumor progression, with Exos acting as critical regulators 31, 32. To investigate the role of Exos derived from acidic PDAC cells in liver metastasis, we established an *in vitro* acidic model. BxPC-3 cells were cultured under stable acidic conditions (pH 6.5) for over five passages to ensure adaptation (**Fig. S1A-G**). Subsequently, Exos were isolated from BxPC-3 cells maintained under acidic and normal conditions (denoted as Exo-A and Exo-N, respectively) and characterized accordingly. TEM and NTA revealed that the isolated Exos had an average diameter of approximately 150 nm **(Fig. S2A-B)**. WB confirmed the expression of exosomal markers (CD9, Tsg101, HSP70) and the absence of the negative marker Calnexin, validating the successful isolation of Exos **(Fig. S1C)**.

**Figure 1:**
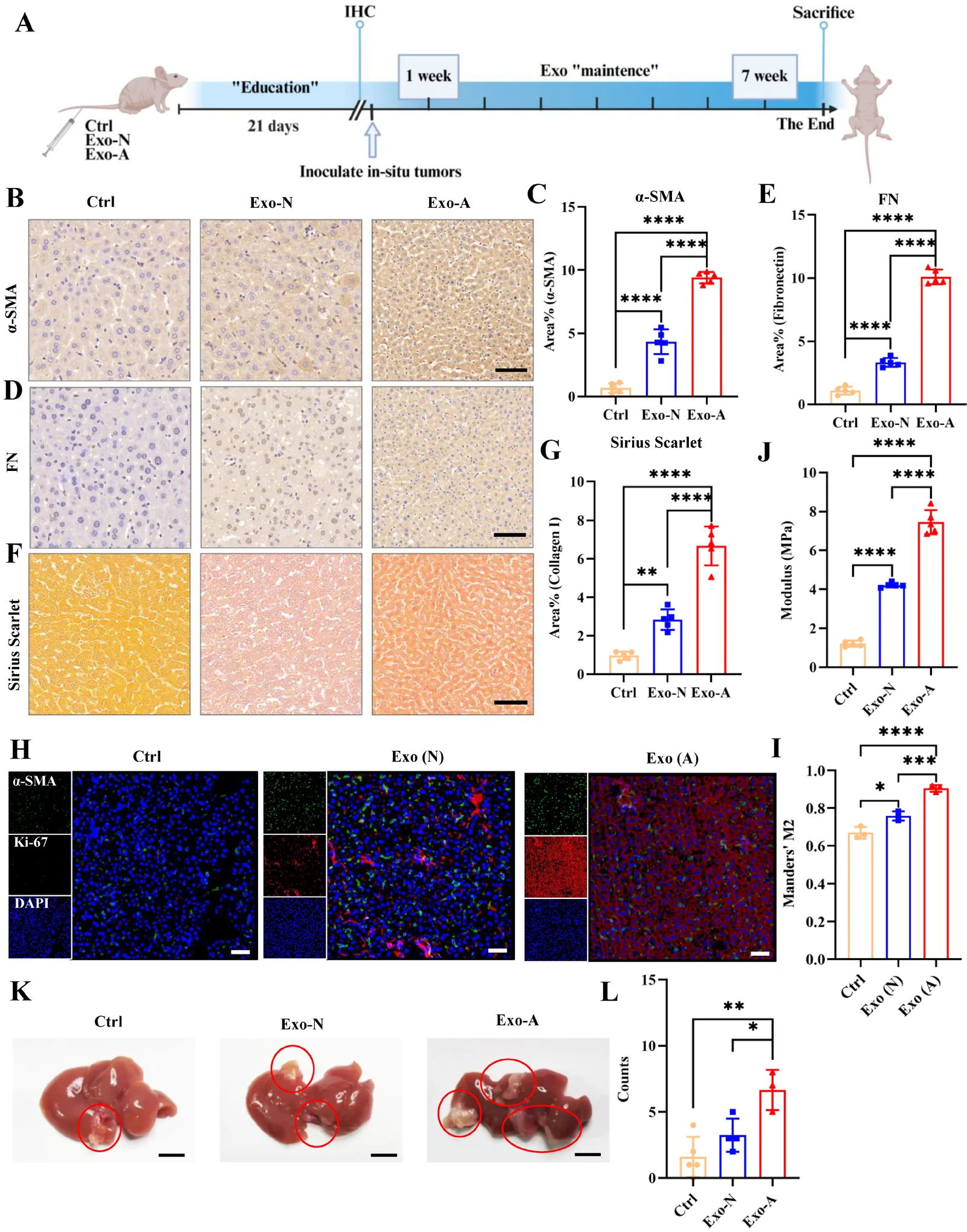
Exo-A promotes PDAC liver metastasis. **(A)** Schematic of the liver “Exos-education” mouse model. **(B-G)** IHC staining and quantitative analysis of α-SMA (C), FN (E), Sirius Scarlet (G) in Exos-educated liver tissue. Scale bar, 100 μm. **(H-I)** IF colocalization staining and quantitative analysis of Ki67 and α-SMA in Exos-educated liver tissue. Scale bar, 100 μm. **(J)** Quantification of ECM stiffness in mouse liver following Exo-A and Exo-N “education.” **(K-L)** *In-situ* transplanted tumor model after “Exos education” (G), and representative images with quantification of PDAC liver metastasis (H). Scale bar, 1 cm. ∗p < 0.05, ∗∗p < 0.01, ∗∗∗p < 0.001, ∗∗∗∗p < 0.0001. Abbreviation: Exos, exosomes; PDAC, pancreatic ductal adenocarcinoma; IHC, Immunohistochemistry; FN, fibronectin; ECM, extracellular matrix; α-SMA, alpha-smooth muscle actin; SD, standard deviation.

The “Exos education” mouse model, a widely utilized method, was employed to evaluate the role of Exos in HSC activation and PDAC liver metastasis 33. Mice were injected with Exo-A or Exo-N via the tail vein for 21 days. Liver tissues were then collected and subjected to IHC analysis to assess HSC activation (α-SMA) and ECM remodeling, indicated by FN and collagen I expression visualized by Sirius Red staining** (Fig. 1A)**. IHC analysis revealed that α-SMA, FN, and collagen I levels were significantly increased in Exo-treated livers, with the most pronounced increase in the Exo-A group **(Fig. 1B-G)**. Consistent with HSC activation, Ki67 expression was also upregulated in Exo-A-treated livers **(Fig. 1H-I)**. To assess changes in liver stiffness induced by Exos, an AFM was used to measure the Young's modulus of the liver after “Exos education.” Exo treatment induced pro-fibrotic changes accompanied by a measurable increase in liver stiffness** (Fig. 1J)**. Next, an orthotopic tumor transplantation model was established to confirm that Exos promote PDAC liver metastasis by activating HSCs **(Fig. 1K)**. The *in vivo* experiments demonstrated that Exos, especially Exo-A, facilitated PDAC liver metastasis **(Fig. 1L)**. These findings confirm that Exos derived from an acidic environment activate HSCs and remodel a pro-fibrotic microenvironment to promote PDAC liver metastasis.

### Exo-A facilitates HSC activation and ECM remodeling *in vitro*

**Figure 2:**
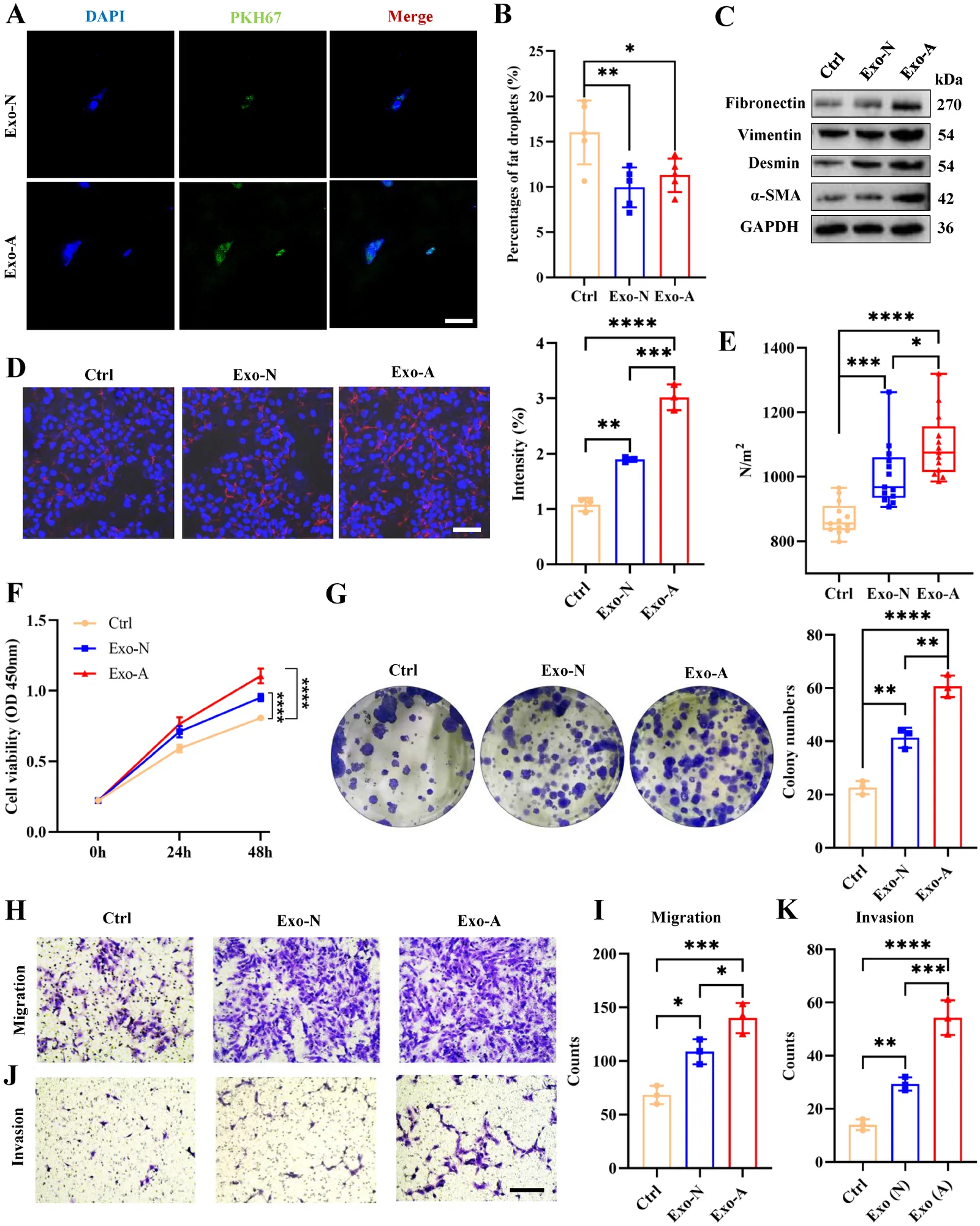
Exo-A promotes HSC activation and ECM remodeling. **(A)** IF assays showing uptake of PKH-67-labeled Exo-A and Exo-N by LX-2 cells. Scale bar, 10 μm. **(B)** Quantification of Oil Red O staining following co-culture of LX-2 cells with Exo-A and Exo-N. **(C)** WB analysis revealing α-SMA, Desmin, Vimentin and FN expression in LX-2 cells treated with Exo-A and Exo-N. **(D)** Representative IF images showing increased collagen I (red) fluorescence intensity following Exo-A and Exo-N stimulation. Scale bar, 50 μm. **(E)** Quantification of ECM stiffness in LX-2 cells after treatment with Exo-A and Exo-N. **(F)** CCK-8 assay measuring the proliferative capacity of LX-2 cells treated with Exo-A and Exo-N. **(G)** Representative images of colony formation assays in LX-2 cells pretreated with Exo-A and Exo-N.** (H-K)** Transwell assays assessing the migration and invasion capacities of LX-2 cells after culturing with Exo-A and Exo-N. Scale bar, 100 μm. ∗p < 0.05, ∗∗p < 0.01, ∗∗∗p < 0.001, ∗∗∗∗p < 0.0001. Abbreviations: WB, western blot; IF, immunofluorescence; ECM, extracellular matrix; FN, fibronectin; α-SMA, alpha-smooth muscle actin; CCK-8, cell counting Kit-8; HSC, hepatic stellate cell.

To investigate the effects of Exos on HSC activation and ECM remodeling *in vitro*, LX-2 cells were directly co-cultured with Exos. Exos were labeled with PKH67 to track their internalization, and IF confirmed efficient uptake by LX-2 cells **(Fig. 2A)**. The loss of lipid droplets, a hallmark of quiescent HSCs transforming into cancer-associated fibroblasts (CAFs), results in negative Oil Red O staining 34. Subsequent experiments showed that Exos, particularly Exo-A, significantly activated LX-2 cells **(Fig. 2B, Fig. S3A)**. Consistent with the activated phenotype, WB revealed that Exo-A upregulated α-SMA, Desmin and Vimentin expression **(Fig. 2C, Fig. S3B)**. To further confirm the fibrotic phenotype, the expression of key ECM components (FN and collagen I) was assessed via WB and IF 35. These results showed that both proteins were upregulated in LX-2 cells following Exo-A treatment **(Fig. 2C-D, Fig. S3B)**. Secreted ECM components collectively create a pro-fibrotic environment and enhance matrix stiffness. AFM confirmed a significant rise in ECM stiffness after LX-2 cells were pre-treated with Exo-A **(Fig. 2E)**. The proliferative capacity of LX-2 cells, assessed using Cell Counting Kit-8 (CCK-8) and colony formation assays, was significantly enhanced by Exo-A **(Fig. 2F and G)**. Transwell assays further revealed that Exo-A markedly promoted LX-2 cell migration and invasion **(Fig. 2H-K)**. These data establish the potent role of Exo-A in driving HSC activation and ECM remodeling *in vitro*.

### Exo-A remodeled the ECM to promote PDAC malignant progression

**Figure 3:**
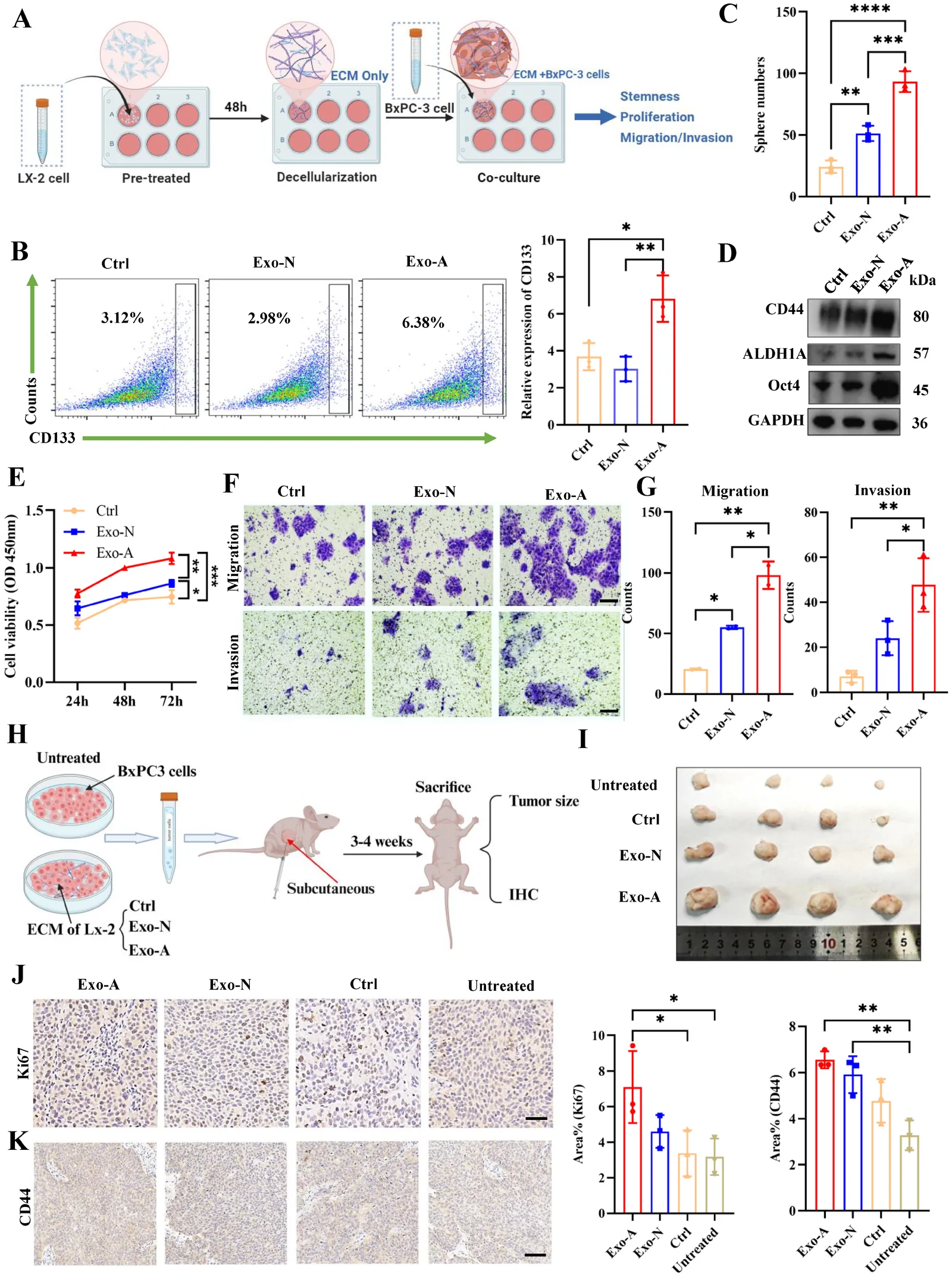
Exo-A-mediated ECM remodeling promotes PDAC stemness. **(A)** Schematic diagram of ECM extraction from LX-2 cells and co-culture with PDAC cells. **(B)** Flow cytometry analysis evaluating the effect of ECM remodeled by LX-2 cells pretreated with Exo-A and Exo-N on PDAC cell stemness. **(C)** Quantitative analysis of tumor-sphere formation of PDAC cells cultured on ECM remodeled by LX-2 cells pretreated with Exo-A and Exo-N. **(D)** WB analysis of CD44, ALDH1A, and Oct4 expression in PDAC cells co-cultured with remodeled ECM by LX-2 cells pretreated with Exo-A and Exo-N. **(E)** CCK-8 assay measuring the proliferative capacity of remodeled ECM on PDAC cells cultured on remodeled ECM. **(F-G)** Transwell assays assessing migration and invasion capacities of PDAC cells cultured on remodeled ECM. Scale bar, 100 μm. **(H)** Schematic of the subcutaneous graft tumor model in mice. **(I)** Representative images of subcutaneous grafted tumors. **(J-K)** IHC staining and quantitative analysis of Ki67 and CD44 in subcutaneous grafted tumors. Scale bar, 100 μm. ∗p < 0.05, ∗∗p < 0.01, ∗∗∗p < 0.001, ∗∗∗∗p < 0.0001. Abbreviations: PDAC, pancreatic ductal adenocarcinoma; WB, western blot; IHC, immunohistochemistry; ECM, extracellular matrix; Exos, exosomes; CCK-8, Cell Counting Kit-8; HSC, hepatic stellate cell

The PDAC TME is characterized by a rigid ECM formed by activated PSCs, which promotes tumor progression *in situ* 36, 37. However, the influence of stiff ECM formed by activated HSCs on metastatic PDAC remains unclear. To investigate this, ECM from differently treated LX-2 cells was extracted and co-cultured with PDAC cells **(Fig. 3A)**. Flow cytometry and tumor spheroid formation assays were performed to assess how the remodeled ECM affects PDAC stemness. ECM derived from Exo-A-activated LX-2 cells significantly increased the CD133**^+^** subpopulation and enhanced sphere-forming capacity **(Fig. 3B-C, Fig. S3C)**. Consistently, WB revealed marked upregulation of stemness markers, including CD44, ALDH1A, and Oct4, in Exo-treated groups, with the strongest effect observed in the Exo-A group **(Fig. 3D, Fig. S3D)**. Notably, ECM remodeled by Exo-A-activated LX-2 cells promoted PDAC cell proliferation, as assessed via CCK-8 and colony formation assays **(Fig. 3E, Fig. S3E)**. Transwell assays further revealed that ECM from LX-2 cells, particularly Exo-A-activated, significantly enhanced PDAC cell migration and invasion **(Fig. 3F-G)**. To examine the association between tumor stemness and tumorigenicity, subcutaneous xenograft models were established **(Fig. 3H)**. ECM from Exo-A-activated LX-2 cells markedly promoted PDAC growth *in vivo* **(Fig. 3I, Fig. S3F)**. Subsequent IHC analysis revealed strong upregulation of CD44, ALDH1A, and Ki67, especially in the Exo-A group **(Fig. 3J-K, Fig. S3G)**. Collectively, these findings indicate that Exo-A is crucial in remodeling the ECM of LX-2 cells to contribute to PDAC malignant progression.

### Exo-A enriches miR-1246 to activate HSCs and remodel stiff ECM

**Figure 4:**
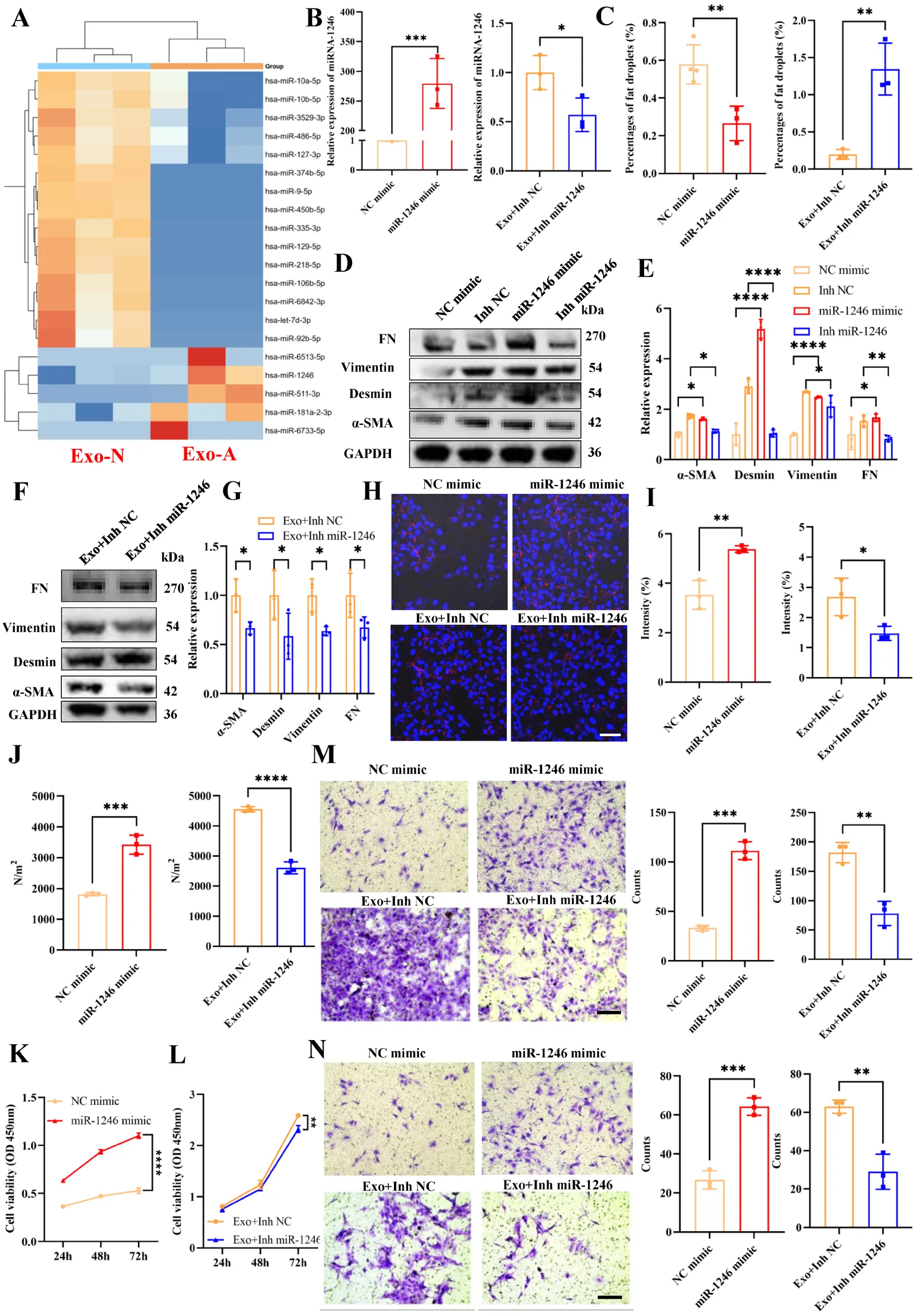
miR-1246 enrichment in Exo-A promotes HSC activation and ECM remodeling. **(A)** MiRNA sequencing revealed differential miRNA expression between Exo-A and Exo-N. **(B)** qRT-PCR analysis of miR-1246 expression following transfection with miR-1246 mimics and inhibitors, with GAPDH as the endogenous control. **(C)** Quantification of Oil Red O staining after miR-1246 overexpression and inhibition in Exo-A-pretreated LX-2 cells. **(D-G)** WB analysis and quantitative analysis of α-SMA, Desmin, Vimentin and FN expression following miR-1246 upregulation/downregulation, and inhibition in Exo-A-pretreated LX-2 cells. **(H-I)** Representative IF images showing increased collagen I (red) fluorescence intensity after miR-1246 overexpression or inhibition in Exo-A-pretreated LX-2 cells. Scale bar, 50 μm. **(J)** The statistical results of ECM stiffness of Lx-2 cells after treating them with miR-1246 mimics and pre-treated with Exo-A. **(K-L)** CCK-8 assay assessing LX-2 cell proliferation. **(M-N)** Transwell assays evaluating LX-2 cell migration (I) and invasion (J) after miR-1246 mimics and inhibition in Exo-A-pretreated cells. Scale bar, 100 μm. ∗p < 0.05, ∗∗p < 0.01, ∗∗∗p < 0.001, ∗∗∗∗p < 0.0001. Abbreviations: HSC, hepatic stellate cell; ECM, extracellular matrix; miRNA, microRNA; qRT-PCR, quantitative reverse transcription polymerase chain reaction; GAPDH, glyceraldehyde-3-phosphate dehydrogenase; α-SMA, alpha-smooth muscle actin; FN, fibronectin; IF, immunofluorescence; CCK-8, Cell Counting Kit-8; LX-2, human hepatic stellate cell line

TDEs mediate the crosstalk between PDAC cells and HSCs, with enriched miRNAs acting as key regulators of this communication 38, 39. To profile their content, exosomal miRNAs from Exo-A and Exo-N were isolated for sequencing. miR-1246 was significantly upregulated in Exo-A, and Kaplan-Meier analysis revealed that high miR-1246 expression correlated with poor PDAC prognosis, highlighting its clinical relevance **(Fig. 4A, Fig. S4A)**. The qRT-PCR of BxPC-3 cells and their corresponding Exos under normal (N) and acidic (A) conditions showed that, although miR-1246 was downregulated in acidic BxPC-3 cells, it was selectively enriched in Exo-A, suggesting active packaging into Exos **(Fig. S4B-C)**. To investigate miR-1246 function, HSCs were transfected with miR-1246 mimics or inhibitors (Inh miR-1246). The qRT-PCR confirmed successful transfection, with miR-1246 overexpressed in the mimics group and reduced in the inhibitor group **(Fig. 4B)**. HSCs activation was evaluated via Oil Red O staining of lipid droplets **(Fig. 4C, Fig. S3D-E)**. WB showed that miR-1246 upregulated α-SMA, Desmin, Vimentin and FN, while its inhibitor partially reversed the pro-fibrotic effects of Exo-A **(Fig. 4D-G)**. Consistently, IF confirmed that miR-1246 similarly increased collagen I expression **(Fig. 4H-I, Fig. S4F)**. Next, we assessed the mechanical properties of the ECM using AFM. miR-1246 overexpression increased ECM stiffness, while its inhibition partially reversed the stiffening induced by Exo-A **(Fig. 4J, Fig. S4G)**. CCK8 and colony formation assays showed that miR-1246 promoted LX-2 cell proliferation, whereas its inhibitor partially rescued the Exo-A-induced proliferation **(Fig. 4K-L, Fig. S4H-J)**. Transwell assays further demonstrated that miR-1246 promoted LX-2 cell migration and invasion, and its inhibitor attenuated the Exo-A-induced effects **(Fig. 4M-N, Fig. S4K)**. Overall, these findings indicate that miR-1246, highly enriched in Exo-A, activates HSCs and remodels ECM, thereby establishing a pro-fibrotic microenvironment.

### MiR-1246 promotes PDAC malignant progression by remodeling the HSC-activated ECM

**Figure 5:**
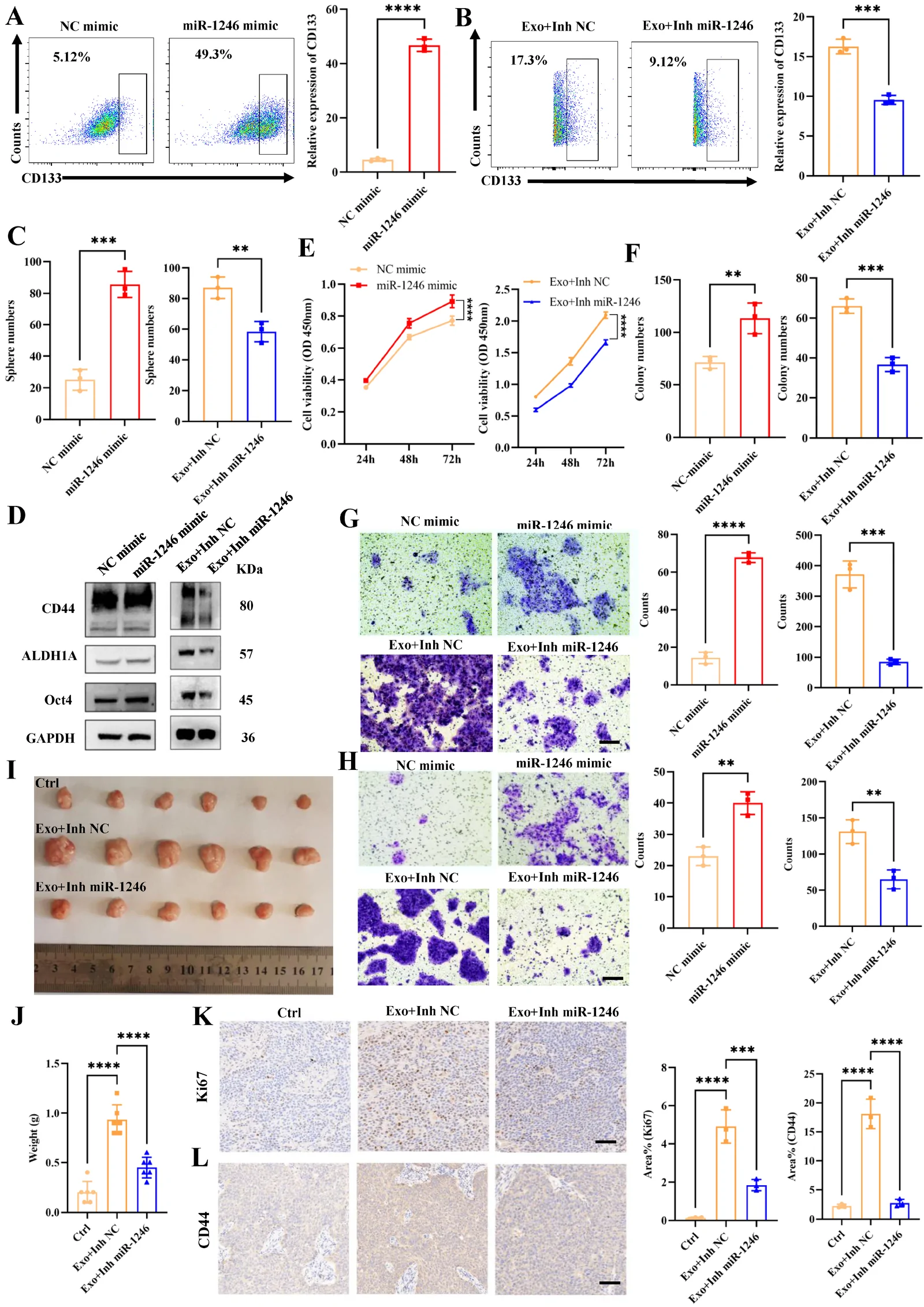
miR-1246 remodels the ECM of HSCs to promote PDAC stemness. **(A-B)** Flow cytometry analysis validating the effect of ECM remodeling by LX-2 cells transfected with miR-1246 mimics and inhibitors and pretreated with Exo-A on PDAC cell stemness. **(C)** Quantification of tumor-sphere formation of PDAC cells cultured on remodeled ECM derived from LX-2 cells overexpressing or silencing miR-1246 following Exo-A pretreatment. **(D)** WB analysis revealing CD44, ALDH1A, and Oct4 expression in PDAC cells co-cultured with remodeled ECM from LX-2 cells transfected with miR-1246 mimics or inhibitors pre-stimulated with Exo-A. **(E)** CCK-8 assay evaluating PDAC cell proliferation on remodeled ECM. **(F)** Representative colony formation assay images of PDAC cells cultured on remodeled ECM. **(G-H)** Transwell assays assessing PDAC cell migration (F) and invasion (G) capacities on remodeled ECM. Scale bar, 100 μm.** (I-J)** Representative images of subcutaneous grafted tumors (I) and quantitative analysis of tumor weights (J). **(K-L)** IHC staining and quantitative analysis of Ki67 and CD44 expression in subcutaneous grafted tumors. Scale bar, 100 μm. ∗p < 0.05, ∗∗p < 0.01, ∗∗∗p < 0.001, ∗∗∗∗p < 0.0001. Abbreviations: CCK-8, cell counting Kit-8; HSC, hepatic stellate cell; PDAC, pancreatic ductal adenocarcinoma; WB, western blot; IF, immunofluorescence; IHC, immunohistochemistry; ECM, extracellular matrix

The remodeled HSC-activated ECM induced malignant progression of PDAC cells, indicating that exosomal miR-1246 mediates these effects by promoting ECM remodeling. Flow cytometry confirmed that miR-1246 mediated the ability of Exo-A to promote the CD133**^+^** subpopulation of PDAC cells through ECM remodeling, as its inhibition partially reversed this effect **(Fig. 5A-B, Fig. S5A)**. Tumor spheroid formation assays further revealed that ECM remodeled by miR-1246-activated LX-2 cells promoted PDAC stem-cell spheroid growth. Moreover, miR-1246 inhibition partially attenuated these Exo-A-induced pro-stemness effects **(Fig. 5C, Fig. S5B-C)**. Consistently, WB analysis demonstrated that the stemness markers CD44, ALDH1A, and Oct4 were significantly upregulated in miR-1246-transfected cells, while miR-1246 inhibition partially reversed their upregulation resulting from Exo-A-induced ECM remodeling **(Fig. 5D, Fig. S5D-G)**. The effect of ECM remodeling by activated LX-2 cells on PDAC cell behavior was then assessed. CCK-8 and colony formation assays revealed that miR-1246-mediated LX-2-remodeled ECM promoted PDAC cell proliferation. Accordingly, the pro-proliferative effect of Exo-A, dependent on this pathway, was reversed by miR-1246 inhibition **(Fig. 5E-F, Fig. S5H-J)**. Transwell assays confirmed that miR-1246 facilitates PDAC cell migration and invasion via LX-2-derived ECM remodeling, an effect dependent on Exo-A, as miR-1246 silencing reversed Exo-A-induced motility **(Fig. 5G-H, Fig. S5K-N)**. Subcutaneous xenograft models were established to assess PDAC stemness and proliferation *in vivo*. Xenograft tumor results demonstrated that Exo-A enhanced PDAC growth through exosomal miR-1246-mediated ECM remodeling in LX-2 cells **(Fig. 5I-J)**. Subsequent IHC analysis confirmed the functional role of miR-1246, showing that its inhibition reduced the tumor expression of CD44, ALDH1A, and Ki67** (Fig. 5K-L, Fig. S5O)**. Collectively, these findings indicate that miR-1246 activates HSCs to remodel the ECM, thereby promoting PDAC malignant progression.

### HuR facilitates miR-1246 enrichment in Exo-A

**Figure 6:**
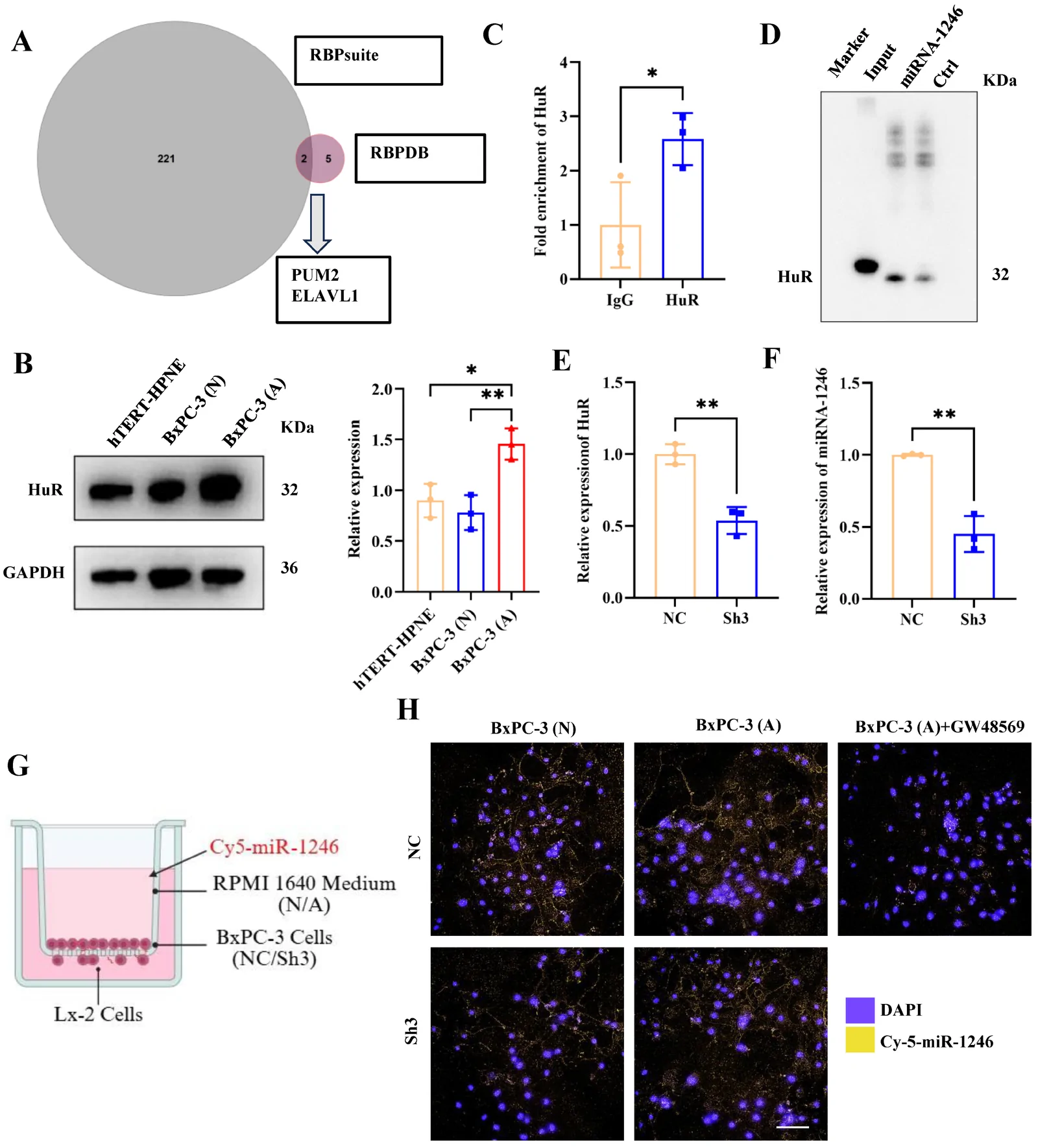
HuR facilitates the enrichment of miR-1246 in Exo-A. **(A)** Predicted RBPs potentially interacting with miR-1246.** (B)** WB analysis and quantitative analysis of HuR expression in hTERT-HPNE and BxPC-3 (N/A) cells. **(C)** RIP assay using anti-HuR antibody (IgG as control) on lysates derived from BxPC-3 (A) cells. miR-1246 levels in immunoprecipitated samples were quantified via qRT-PCR and expressed as a percentage of input (% input).** (F)** RNA pull-down assay assessing HuR expression in samples precipitated with miRNA-1246. **(E)** qRT-PCR analysis revealing HuR expression following shRNA plasmid transfection, with GAPDH as the endogenous control. **(F)** qRT-PCR quantification of exosomal miR-1246 from NC and Sh3 groups, with U6 as the endogenous control. **(G)** Co-culture model of LX-2 cells with BxPC-3 (N/A) cells pre-transfected with sh-HuR and Cy5-miR-1246 (red); nuclei were counterstained with DAPI (blue). **(H)** IF imaging showing red fluorescent signals in LX-2 cells. Scale bar, 100 μm. ∗p < 0.05, ∗∗p < 0.01. Abbreviations: HuR, human antigen R; RBP, RNA-binding protein; WB, western blot; hTERT-HPNE, human telomerase reverse transcriptase-immortalized pancreatic ductal epithelial cells; BxPC-3, human pancreatic ductal adenocarcinoma cell line; RIP, RNA immunoprecipitation; qRT-PCR, quantitative reverse transcription polymerase chain reaction; GAPDH, glyceraldehyde-3-phosphate dehydrogenase; shRNA, short hairpin RNA; NC, negative control; Cy5, cyanine 5 fluorescent dye; DAPI, 4′,6-diamidino-2-phenylindole; IF, immunofluorescence; LX-2, human hepatic stellate cell line; N/A, normoxic condition

RBPs mediate the selective enrichment of miRNAs into Exos 40. Despite the marked enrichment of miR-1246 in Exo-A, the mechanism underlying its selective packaging remains unclear. To detect miR-1246-specific RBPs, data from the RBP-specificity database (RBPDB, http://rbpdb.ccbr.utoronto.ca/) were integrated with RBP site predictions from RBPsuite (http://www.csbio.sjtu.edu.cn/bioinf/RBPsuite/). The intersection of the two databases revealed two candidate RBPs: Pumilio RNA Binding Family Member 2 (PUM2) and Embryonic Lethal, Abnormal Vision, Drosophila-Like 1 (ELAVL1, known as HuR)** (Fig. 6A)**. Specifically, HuR participates in miR-1246 enrichment in gastric cancer (GC) TDEs 41. WB analysis demonstrated significant HuR upregulation in BxPC-3 cells compared to that in hTERT-HPNE, particularly under acidic conditions **(Fig. 6B)**. The interaction between HuR and miR-1246 was assessed using miRNA pull-down and RBP immunoprecipitation (RIP) assays, verifying their direct binding **(Fig. 6C and D)**. To determine whether HuR was required for miR-1246 enrichment into TDEs, HuR was knocked down in acidic BxPC-3 cells. Subsequent qRT-PCR analysis showed that exosomal miR-1246 levels were significantly reduced following HuR knockdown **(Fig. 6E and F)**. To visualize the role of HuR in enriching exosomal miR-1246, a co-culture system was established, enabling direct comparison between control and HuR-silenced BxPC-3 cells (both transfected with Cy5-labeled miR-1246) in the upper chamber, with LX-2 cells in the lower chamber **(Fig. 6G)**. IF results confirmed that HuR was required for the efficient exosomal delivery of miR-1246 to HSCs, as its knockdown markedly reduced the enrichment. **(Fig. 6H)**. These observations indicate that HuR is the critical RBP regulating miR-1246 loading into TDEs.

### MiR-1246 targets WASF3 to promote HSC activation and remodel stiff ECM

**Figure 7:**
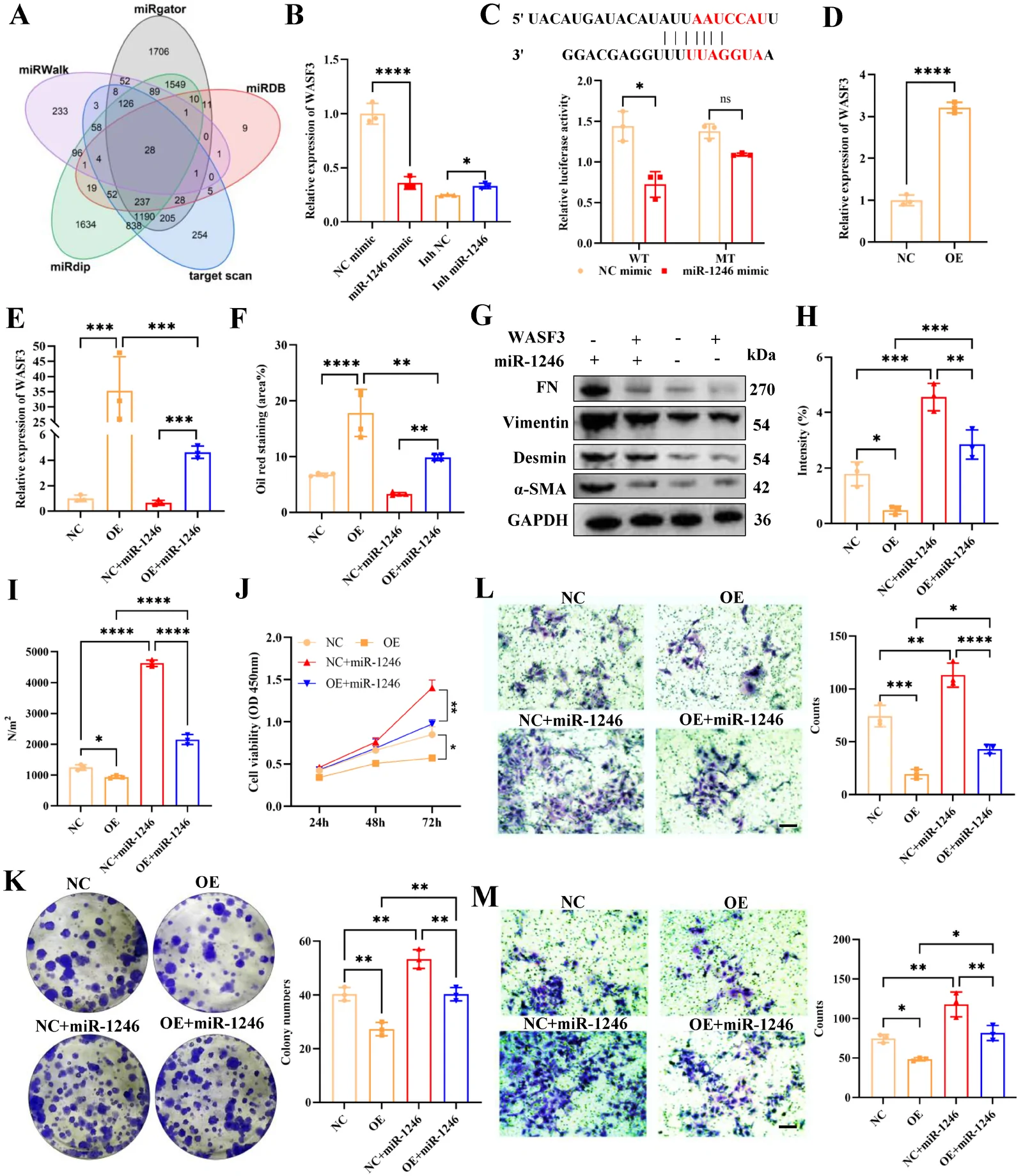
miR-1246 targets WASF3 to promote HSC activation and ECM remodeling. **(A)** Venn diagram showing overlapping target genes of miR-1246 predicted by five bioinformatic algorithms (miRDB, Targetscan, miRWalk, miRgator, miR-DIP). **(B)** qRT-PCR analysis of WASF3 expression in LX-2 cells following miR-1246 overexpression or inhibition, with GAPDH as the endogenous control. **(C)** Predicted binding sites of miR-1246 within the 3′UTR of WASF3 and dual-luciferase reporter assay showing relative luciferase activity.** (D)** qRT-PCR analysis of WASF3 expression in LX-2 cells transfected with NC/OE plasmids, using GAPDH as the endogenous control. **(E)** qRT-PCR validation of WASF3 expression (with GAPDH as control) in LX-2 cells pretreated with NC or OE plasmids followed by miR-1246 transfection. **(F)** Quantification of Oil Red O staining following miR-1246 transfection in LX-2 cells pretreated with WASF3 NC/OE plasmids. **(G)** WB analysis of α-SMA, Desmin, Vimentin and FN expression in LX-2 cells transfected with miR-1246 after pretreatment with WASF3 NC/OE plasmids. **(H)** Representative IF images showing increased Collagen I (red) fluorescence intensity following miR-1246 transfection in LX-2 cells pretreated with WASF3 NC/OE plasmids. Scale bar, 50 μm. **(I)** Statistical analysis of ECM stiffness in LX-2 cells treated with WASF3 NC/OE plasmids. **(J)** CCK-8 assay evaluating the proliferative capacity of LX-2 cells transfected with miR-1246 following WASF3 NC/OE plasmid pretreatment. **(K)** Representative colony formation assay images of LX-2 cells transfected with miR-1246 after WASF3 NC/OE plasmid pretreatment. **(L-M)** Transwell assays assessing LX-2 cell migration and invasion capacity. Scale bar, 100 μm. ∗p < 0.05, ∗∗p < 0.01, ∗∗∗p < 0.001, ∗∗∗∗p < 0.0001. Abbreviations: 3′UTR, 3′ untranslated region; qRT-PCR, quantitative reverse transcription polymerase chain reaction; WASF3, Wiskott-Aldrich syndrome protein family member 3; WB, western blot; IF, immunofluorescence; ECM, extracellular matrix; FN, fibronectin; α-SMA, alpha-smooth muscle actin; NC, negative control; OE, overexpression; CCK-8, Cell Counting Kit-8; HSC, hepatic stellate cell

To examine the mechanism of miR-1246-mediated LX-2 cell activation, a multi-database screening was performed using miRWalk, miRDB, miRdip, TargetScan, and miRgator to predict its target genes. Bioinformatic screening revealed 26 potential target genes, with the tumor suppressor WASF3 being the most significantly affected candidate **(Fig. 7A, Fig. S6A)**. WASF3, a downstream effector of Rac1, was associated with the actin cytoskeleton. In the TCGA cohort, high WASF3 expression correlated with poorer overall survival in patients with PDAC compared with low expression (**Fig. S6B**). WASF3 expression was significantly lower in BxPC-3 cells than in hTERT-HPNE cells under normal and acidic conditions (**Fig. S6C**). Next, miR-1246 regulation of WASF3 in LX-2 cells was examined. qRT-PCR and flow cytometry analyses confirmed that miR-1246 suppressed WASF3 expression, while miR-1246 inhibition attenuated the Exo-A-mediated WASF3 downregulation **(Fig. 7B, Fig. S6D)**. To experimentally validate miR-1246 targeting of WASF3, a dual-luciferase reporter assay was performed, which confirmed that miR-1246 binds directly to the WASF3 3'-UTR, suppressing its expression** (Fig. 7C)**. Following qRT-PCR confirmation that miR-1246 suppressed WASF3, WASF3-overexpressing LX-2 cells were generated, and rescue experiments were performed to examine its role in miR-1246-promoted HSC activation **(Fig. 7D)**. qRT-PCR confirmed that miR-1246 suppressed WASF3 expression **(Fig. 7E)**. Subsequently, Oil Red O staining assay was employed to assess the role of the miR-1246-WASF3 axis in HSC activation. WASF3 overexpression resulted in abundant lipid droplets, indicating an inactive HSC state. However, rescue experiments demonstrated that miR-1246 partially reversed this WASF3-mediated inactivation of LX-2 cells **(Fig. 7F, Fig. S6E)**. Consistently, WB revealed that WASF3 overexpression attenuated α-SMA, Desmin, Vimentin and FN expression, which was partially restored by miR-1246 **(Fig. 7G, Fig. S6F)**. IF also confirmed that miR-1246 enhanced collagen I expression by targeting WASF3, contributing to a pro-fibrotic ECM **(Fig. 7H, Fig. S6G)**. To further confirm the involvement of WASF3 downregulation in HSC activation, LX-2 cells were exposed to TGF-β for 48 hours. Western blot analysis was performed to assess the expression levels of WASF3, HSC activation markers (including α-SMA, Desmin, and Vimentin), and FN. The results showed that TGF-β significantly downregulated WASF3 expression, while it activated LX-2 cells and upregulated FN expression **(Fig. S6H-I)**. Additionally, following the transfection of WASF3 silencing plasmids, it was found that WASF3 knockdown significantly activated LX-2 cells **(Fig. S6J-M)**. These results suggested that WASF3 silencing may serve as a potential marker of HSC activation. To determine how the miR-1246-WASF3 axis influences ECM stiffness, Young's modulus was evaluated using AFM. The suppressive effect of WASF3 on ECM stiffness was attenuated and restored via miR-1246 overexpression **(Fig. 7I)**. To determine whether miR-1246 modulates HSC behavior by targeting WASF3, CCK-8 and colony formation assays were performed. These functional assays demonstrated that miR-1246 promoted LX-2 cell proliferation and colony formation by suppressing WASF3, thereby relieving its inhibitory effect on these phenotypes **(Fig. 7J-K)**. Furthermore, transwell assays revealed that miR-1246 enhanced LX-2 cell migration and invasion through the same WASF3 silencing mechanism **(Fig. 7L-M)**. Collectively, these results demonstrate that miR-1246 targets WASF3 to modulate the pro-fibrotic ECM in LX-2 cells.

### MiR-1246 promotes PDAC progression by targeting WASF3 to remodel pro-fibrotic ECM

**Figure 8:**
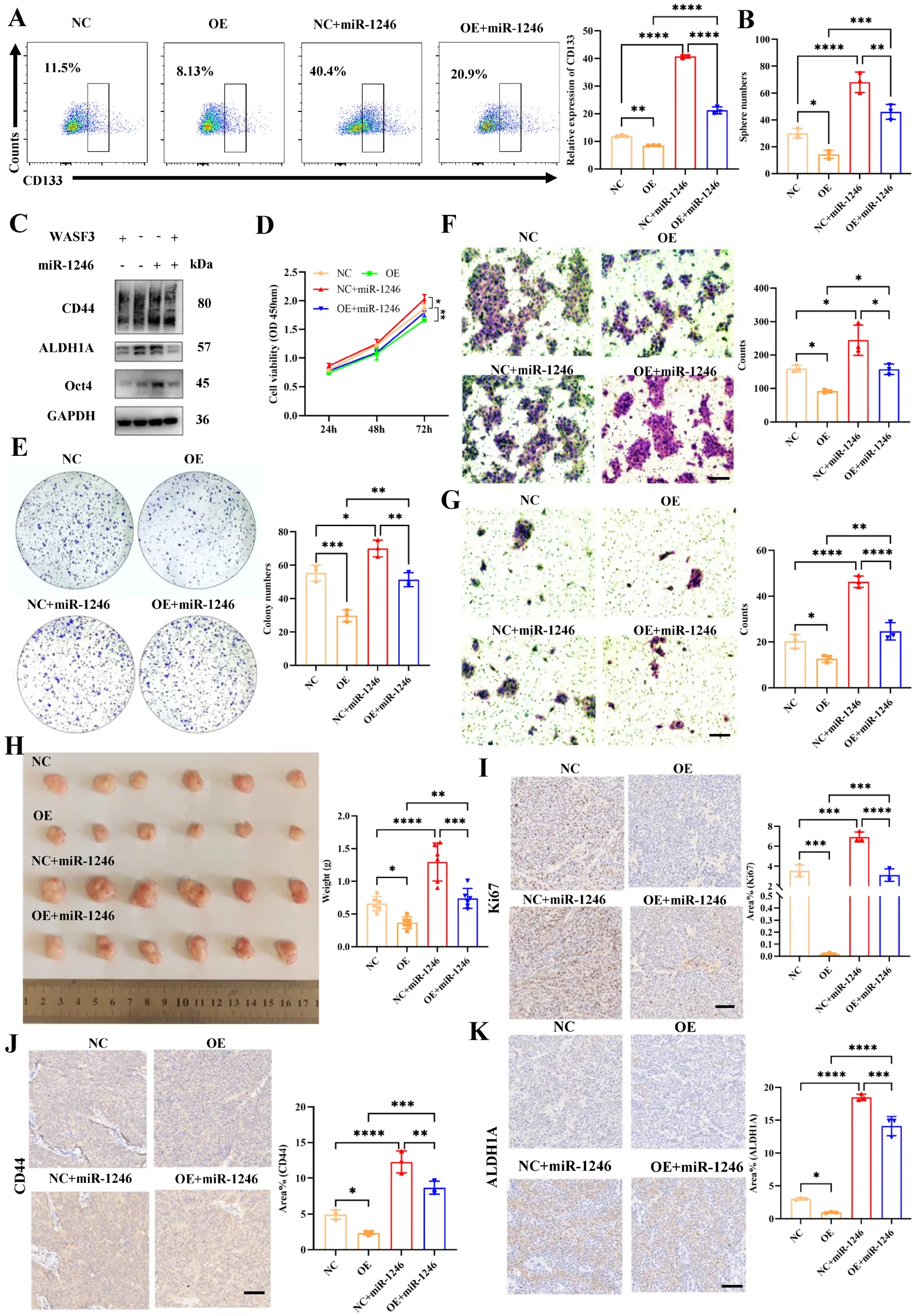
miR-1246 targets WASF3 to remodel the ECM and promote PDAC stemness. **(A)** Flow cytometry analysis validating that miR-1246 targets WASF3 to remodel the ECM of LX-2 cells and induce PDAC cell stemness. **(B)** Quantification of tumor-sphere formation by PDAC cells cultured on ECM remodeled by LX-2 cells overexpressing miR-1246 and pre-transfected with WASF3 NC/OE plasmids. Scale bar, 100 μm. **(C)** WB analysis revealing the expression of CD44, ALDH1A, and Oct4 in PDAC cells co-cultured with remodeled ECM from LX-2 cells pretreated with WASF3 NC/OE plasmids. **(D)** CCK-8 assay evaluating the proliferative capacity of PDAC cells cultured on remodeled ECM. **(E)** Representative colony-formation images of PDAC cells cultured on remodeled ECM. **(F-G)** Transwell assays assessing migration (F) and invasion (G) capacities of PDAC cells cultured on remodeled ECM. Scale bar, 100 μm.** (H)** Representative images and quantification of subcutaneous grafted tumors. **(I-K)** IHC analysis and statistical evaluation of Ki67, CD44, and ALDH1A expression in subcutaneous grafted tumors. Scale bar, 100 μm. ∗p < 0.05, ∗∗p < 0.01, ∗∗∗p < 0.001, ∗∗∗∗p < 0.0001. Abbreviations: CCK-8, cell counting Kit-8; WASF3, Wiskott-Aldrich syndrome protein family member 3; PDAC, pancreatic ductal adenocarcinoma; WB, western blot; IHC, immunohistochemistry; ECM, extracellular matrix; NC, negative control; OE, overexpression

While the above results showed that miR-1246 activated LX-2 cells and remodeled the ECM by targeting WASF3, whether the remodeled ECM influenced PDAC progression remains unclear. Consistently, flow cytometry and spheroid formation assays revealed that miR-1246 restored both the CD133^+^ subpopulation and sphere-forming capacity by reversing the inhibitory effects of ECM produced by WASF3-overexpressing LX-2 cells **(Fig. 8A and B, Fig. S6N)**. WB also confirmed that WASF3 overexpression suppressed stemness markers, including CD44, ALDH1A, and Oct4, while miR-1246 restored their expression** (Fig. 8C, Fig. S6O)**. To assess how the remodeled ECM contributes to PDAC progression, functional assays were performed. CCK-8 and colony formation assays confirmed that miR-1246 enhanced PDAC proliferation and clonogenicity by targeting WASF3 and inducing ECM remodeling **(Fig. 8D and E)**. Similarly, transwell assays demonstrated that WASF3 overexpression partially attenuated the pro-metastatic effects of the miR-1246-remodeled ECM **(Fig. 8F and G)**. Subcutaneous xenograft models were established to validate the functional effect of stemness *in vivo*. Pre-treating PDAC cells with the miR-1246-remodeled ECM substantially enhanced tumorigenicity and growth **(Fig. 8H)**. Furthermore, IHC analysis of tumors confirmed that miR-1246 overexpression reversed the suppressive effects of WASF3 overexpression on CD44, ALDH1A, and Ki67 **(Fig. 8I-K)**. Collectively, both* in vitro* and *in vivo* results revealed that miR-1246 promotes PDAC progression through a cascade involving WASF3 targeting, LX-2 cell activation, and stiff ECM remodeling.

### TDE miR-1246 contributes to HSC activation and PDAC liver metastasis via WASF3 targeting and modulating the PI3K/Akt pathway

**Figure 9:**
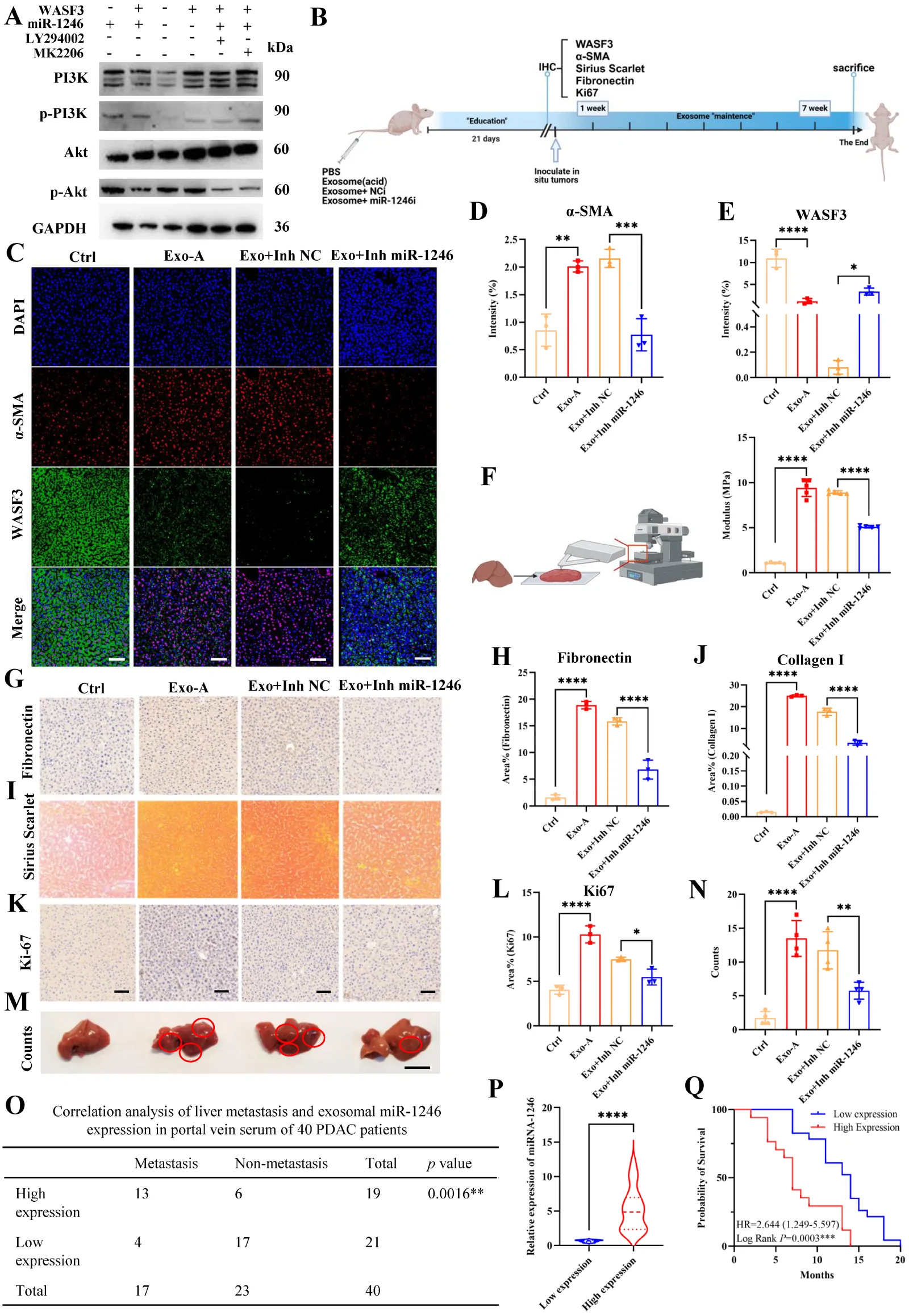
TDE miR-1246 targets WASF3 to promote PDAC liver metastasis. **(A)** WB analysis of protein expression in the PI3K/AKT signaling pathway in LX-2 cells transfected with miR-1246 and pretreated with WASF3 NC/OE plasmids (including PI3K inhibitor-LY294002, and Akt inhibitor-MK2206). **(B)** Schematic diagram of the rescue experiment using the “Exos education” model. **(C-E)** IF analysis and quantification of α-SMA and WASF3 in TDE rescue-educated liver. Scale bar, 100 μm. **(F)** Quantification of ECM stiffness in mouse livers after Exo-A rescue “education.”** (G-J)** IHC analysis and quantification of FN (G, H), Sirius Scarlet staining (I, J) in Exo-A rescue-educated liver. Scale bar, 100 μm.** (K, L)** IHC analysis and quantification of Ki67 in educated livers. **(M-N)** Representative images and quantification of PDAC liver metastasis after “Exos education,” establishing an in-situ transplanted tumor.** (O)** Correlation between liver metastasis and exosomal miR-1246 expression in portal-vein serum from 40 PDAC patients.** (P)** Expression levels of exosomal miR-1246 in portal-vein serum from 40 PDAC patients, with U6 as the endogenous control (n = 3). **(Q)** Kaplan**-**Meier survival curves stratified by low/high exosomal miR-1246 expression in 40 PDAC patients. Scale bar, 1 cm. ∗p < 0.05, ∗∗p < 0.01, ∗∗∗p < 0.001, ∗∗∗∗p < 0.0001. Abbreviations: TDEs, tumor-derived exosomes; WASF3, Wiskott-Aldrich syndrome protein family member 3; PDAC, pancreatic ductal adenocarcinoma; WB, western blot; IF, immunofluorescence; IHC, immunohistochemistry; ECM, extracellular matrix; Exos, exosomes; FN, fibronectin; α-SMA, alpha-smooth muscle actin; NC, negative control; OE, overexpression

To examine the key downstream pathways altered in WASF3-overexpressing cells, transcriptome sequencing (RNA-seq) was conducted, and significant pathways were screened. Gene Ontology enrichment analysis revealed that differentially expressed genes were significantly enriched in ECM organization, receptor complexes, and ECM structural constituents. Particularly, RNA sequencing detected a marked downregulation of the PI3K/Akt pathway in WASF3-overexpressing cells **(Fig. S7A-D)**. Corroborating these findings, WB analysis demonstrated that miR-1246 activated the PI3K/Akt pathway by targeting WASF3 **(Fig. 9A, S7E)**. Furthermore, inhibition of the PI3K-Akt pathway significantly reverses the expression of α-SMA and Desmin, as well as ECM components (including fibronectin and collagen I), confirming that PI3K and Akt inhibitors suppress LX-2 activation and ECM remodeling **(Fig. S7F-G)**.

To determine the role of exosomal miR-1246 in PDAC liver metastasis *in vivo*, specifically its targeting of WASF3 in HSCs to remodel pro-fibrotic ECM, a liver “Exos education” model was established. Briefly, Exos isolated from four treatment groups (Ctrl, Exo-A, Exo-A**+**Inh-NC, and Exo-A**+**Inh-miR-1246) were administered to mice via tail vein injection over 21 days to simulate the hepatic PMN *in vivo* **(Fig. 9B)**. Mice were sacrificed, and livers were collected for analysis. IF was performed to characterize α-SMA and WASF3 localization and expression in HSCs, revealing their co-localization. Specifically, IF analysis showed that miR-1246 inhibition downregulated α-SMA and upregulated WASF3, indicating reversal of Exo-A-induced WASF3 silencing and HSC activation **(Fig. 9C-E)**. To quantify the effect of miR-1246 inhibition on ECM stiffness, AFM was used to measure the liver Young's modulus, demonstrating that miR-1246 inhibitors reversed the Exo-A-induced increase in liver stiffness** (Fig. 9F)**. Subsequent IHC analyses were conducted to evaluate FN expression, collagen I (visualized by Sirius Red staining), and Ki67. Administration of the miR-1246 inhibitors suppressed FN and collagen I expression **(Fig. 9G-J)**. These findings revealed that TDE-derived miR-1246 activated HSCs to form a stiff microenvironment. Ki67 staining also showed that miR-1246 targeted WASF3 to promote HSC proliferation *in vivo* **(Fig. 9K and L)**. To assess the contribution of ECM stiffness to PDAC liver metastasis, in-situ transplantation models were established after the mice underwent a 21-day liver “Exos education” protocol. The results demonstrated that TDE-derived miR-1246 contributes to PDAC liver metastasis by targeting WASF3 and reshaping the fibrotic microenvironment. **(Fig. 9M and N)**. These findings indicate that, through WASF3-mediated HSC activation, TDE-derived miR-1246 remodels the ECM into a stiff and pro-metastatic niche, thereby facilitating PDAC liver metastasis. Finally, clinical information was collected from 40 patients with PDAC, including with and 23 without liver metastasis **(Table 2)**. The results showed that patients with liver metastasis exhibited higher exosomal miR-1246 expression, indicating a correlation between liver metastasis and exosomal miR-1246 levels **(Fig. 9O and P)**. To assess the prognostic value of miR-1246, Kaplan-Meier survival analysis was performed. Patients with high exosomal miR-1246 expression exhibited poorer prognosis and significantly shorter survival **(Fig. 9Q)**.

## Discussion

Tumor metastasis remains a major contributor to poor prognosis in patients with PDAC, and the formation of hepatic PMN represents a critical initiating event in this process 42. TDEs are key mediators of hepatic PMN formation, creating a favorable microenvironment for subsequent metastasis 43. Previous studies demonstrate that exosomal proteins, including MIF and the CD44v6/C1QBP complex, activate HSCs and remodel the ECM, thereby promoting hepatic PMN formation 44, 45. Notably, exosomal noncoding RNAs (ncRNAs) play pivotal roles in promoting PDAC liver metastasis by constructing a stiff and immunosuppressive microenvironment 46. Among exosomal ncRNAs, TDE-derived miRNAs serve as essential mediators of intercellular communication, regulating gene expression and influencing cellular behavior 47. miR-1246, transcribed from the MIR1246 gene, is regulated by the tumor suppressor p53 48, 49. Despite the established specificity of miR-1246 in gastrointestinal tumors, its mechanistic contribution to metastasis progression remains unclear and warrants further investigation.

Recent evidence demonstrates that *Fusobacterium nucleatum* (Fn) stimulates tumor cells to secrete miR-1246-rich Exos, which are subsequently taken up by uninfected cells, promoting metastatic behavior 50. Another study reveals that TDE miR-1246 suppresses insulin-induced gene 1 expression, increasing free cholesterol synthesis and activating the TLR4/NF-κB/TGF-β signaling pathway in HSCs, ultimately promoting tumor metastasis 51. In this study, exosomal miR-1246 contributes to HSC activation and stiff ECM remodeling through WASF3 silencing. RBPs are key regulators of miRNA biogenesis and their selective enrichment into Exos. Previous literature reports that serine- and arginine-rich splicing factor 1 (SRSF1) binds a specific motif within the miR-1246 sequence, mediating its selective incorporation into Exos 52. Additionally, HuR is implicated in the packaging of miR-1246 into serum TDEs 41, and a CD9-HuR fusion strategy has been developed as a tool for targeting miRNA encapsulation within Exos 53. Collectively, RBPs are emerging as central regulator of the pro-metastatic TDE-mediated functions 54. Consistently, this study also identifies HuR as the primary regulator mediating miR-1246 loading into Exos under acidic microenvironments. Furthermore, the critical role of miR-1246 in HSC activation and promoting liver metastasis was confirmed *in vivo* and* in vitro*.

ECM remodeling is a hallmark of the PMN establishment, with activated HSCs generating a fibrotic liver microenvironment that facilitates metastasis in a secondary environment 44, 55. The remodeled ECM provides critical attachment sites for CTCs and serves as a supportive niche for infiltrating macrophages and myeloid-derived suppressor cells 3, 56. PDAC organoids in high-stiffness gelatin matrices exhibit a more compact morphology and enhance stem-like properties relative to those cultured in softer matrices 57. Furthermore, under high-stiffness conditions, CAFs transfer large quantities of mitochondria to tumor cells through tunneling nanotubes, enhancing their migratory capacity 58. In this study, WB and IF results show that exosomal miR-1246 upregulated collagen I and FN expression, thereby inducing ECM remodeling. Specifically, AFM analyses revealed that HSC activation directly increased ECM stiffness. To examine the effect of stiff ECM on tumor cell behavior, a co-culture system of PDAC cells with HSC-derived ECM was established. Flow cytometry and WB analyses revealed that ECM from activated HSCs enhanced PDAC stemness. A subcutaneous graft tumor model also indicated that ECM remodeling promoted PDAC stemness and tumorigenicity.

WASF3, a key actin cytoskeleton-associated gene related to tumor cell motility 59, 60, promotes ECM remodeling by regulating the expression and activity of key MMPs 24. Although WASF3 modulates tumor cell migration and invasion, its role in stromal cells, particularly in activating HSCs to remodel the ECM and promote tumor progression, remains unclear. WASF3-mediated functions depend on NF-κB and PI3K/Akt signaling 61. The p85 regulatory subunit of PI3K binds to the C-terminal SH2 domain of WASF3, mediating its function 62. PI3K signaling also plays a crucial role in fibroblast motility 63. Extensive studies on WASF3, miR-1246, and PI3K/Akt signaling show their vital roles in regulating HSC activation following miR-1246 targeting of WASF3 64, 65. In this study, WASF3 was validated as a direct downstream target of miR-1246 in HSCs through qPT-PCR and dual-luciferase reporter assays. Specifically, IF of liver tissue from “Exos-educated” mice showed that WASF3 downregulation co-localized with α-SMA, consistent with miR-1246-mediated regulation. Furthermore, mRNA transcriptome sequencing across treatment groups revealed the key role of PI3K/Akt signaling pathway in mediating HSC activation upon WASF3 silencing.

However, this study has some limitations. First, although TDE-derived miR-1246 shows promise as a diagnostic biomarker for PDAC liver metastasis, additional clinical validation is required to confirm its diagnostic value. Future research should also focus on developing a multi-marker panel incorporating miR-1246 to improve early detection of PDAC liver metastasis. Second, while this study shows that ECM remodeling promotes stem-like properties in tumor cells, future investigation is needed to elucidate the underlying mechanisms. Evidence indicates that ECM remodeling enhances tumor cell stemness by activating the yes1-associated transcriptional regulator (YAP)-transcriptional coactivator with PDZ-binding motif (TAZ) signaling pathway, suggesting a potential mechanism 66, 67. Third, future studies should extend these findings by investigating additional PMN features, including immunosuppression, metabolic reprogramming, and the crosstalk between the PMN and the primary TME.

In conclusion, miR-1246 was enriched in Exos under acidic conditions through HuR-mediated regulation. Exosomal miR-1246 targets WASF3 to induce the stiff ECM via HSC activation, establishing the hepatic PMN to promote PDAC liver metastasis. Elevated expression of exosomal miR-1246 and its target gene WASF3 represents promising biomarkers for early diagnosis, prognosis assessment, and therapeutic development in PDAC patients with liver metastasis.

## Supplementary Material

Supplementary figures and tables.

## Figures and Tables

**Table 1:** List of primers for RT-qPCR.

	Primers
miR-1246-RT	GTCGTATCCAGTGCAGGGTCCGAGGTATTCGCACTGGATACGACCCTGCT
U6-F	CTCGCTTCGGCAGCACA
U6-R	AACGCTTCACGAATTTGCGT
miR-1246F	CGCGTGGAGAGAGAAAAGAGA
miR-1246-R	AGTGCAGGGTCCGAGGTATT
GAPDH-F	GTATTGGGCGCCTGGTCACC
GAPDH-R	CGCTCCTGGAAGATGGTGATGG
WASF3-F	GGGATTACCAGCGAACTTGA
WASF3-R	AATCCAGCTGGGTGACTTTG

**Table 2:** Clinical data of 40 PDAC patients with or without liver metastasis.

Variables	Liver metastasis (n = 17)	Non-liver metastasis (n = 23)	p value
Ages			0.676
≥ 65	10	12	
< 65	7	11	
Sex			0.554
Male	8	13	
Female	9	10	
BMI			0.218
≥ 24.0	7	14	
< 24	10	9	
Diabetes			0.443
Yes	4	8	
No	13	15	
CA199			0.717
High	15	20	
Low	2	3	
Vascular invasion			0.003**
Yes	14	8	
No	3	15	
Perineural invasion			0.005**
Yes	12	6	
No	5	17	
Lymph node			0.214
metastasis	16	17	
T Stage			0.088
1-2	5	13	
3-4	12	10	
N Stages			0.214
0	1	6	
1-2	16	17	
Yes	1	6	
No			
M Stages			0.000****
M0	0	23	
M1	17	0	

## Data Availability

The data that support the findings of this study are available from the corresponding author upon reasonable request.

## Abbreviations

Akt: Serine/threonine protein kinase
AFM: atomic force microscopy
CAFs: cancer-associated fibroblasts
CCK-8: Cell counting Kit-8
CCNG2: Cyclin G2
CTCs: circulating tumor cells
CRC: colorectal carcinoma
DAB: diaminobenzidine
ECM: extracellular matrix
EVs: extracellular vesicles
ELAVL1: (Embryonic Lethal, Abnormal Vision, Drosophila)-Like 1
Exos: Exosomes
FN: Fibronectin
Fn: *Fusobacterium nucleatum*
GC: gastric cancer
GEM: gemcitabine
GI: gastrointestinal
HSCs: Hepatic stellate cells
HSP70: heat shock protein 70
HuR: Human Antigen R
IF: Immunofluorescence
IHC: Immunohistochemistry
INSIG1: insulin induced gene 1
miRNAs: microRNAs
MAPK: mitogen-activated protein kinase
MDSCs: myeloid-derived suppressor cells
MMPs: matrix metalloproteinases
NC: negative control
ncRNAs: noncoding RNAs
NF-κB: nuclear factor kappa B
NTA: nanoparticle tracking assay
OE: overexpression
PDAC: Pancreatic ductal adenocarcinoma
PI3Kγ: phosphatidylinositol 3-kinase subunit Gamma
PFA: paraformaldehyde
PMN: pre-metastatic niche
PSCs: pancreatic stellate cells
PTEN: phosphatase and tensin homolog
PUM2: Pumilio RNA Binding Family Member 2
qRT-PCR: Quantitative reverse transcriptase PCR
Rac1: Ras-related C3 botulinum toxin substrate 1
RBP: RNA binding protein
RIP: RBP immunoprecipitation
SD: standard deviation
SRSF: serine and arginine rich splicing factor 1
TAZ: transcriptional coactivator with PDZ-binding motif
TDEs: tumor-derived exosomes
TME: tumor microenvironment
TSG101: tumor susceptibility gene 101
WASF3: WASP Family Member 3
WASP: Wiskott-Aldrich syndrome protein
WB: Western blotting
YAP: yes1 associated transcriptional regulator
