# Supplementary material for: The Acidic Exosomal miR-1246/WASF3 Axis Regulates Hepatic Stellate Cell Activation and Stiff ECM Remodeling to Promote Pancreatic Ductal Adenocarcinoma Liver Metastasis
Source: Int J Biol Sci. 2026 Aug 21;22(13):7358–79. doi: 10.7150/ijbs.132772 (PMC13541008; doi:10.7150/ijbs.132772)
Supplement: Supplementary file 1 [file ijbsv22p7358s1.pdf]

The Acidic Exosomal miR-1246/WASF3 Axis Regulates Hepatic Stellate Cell Activation and Stiff ECM Remodeling to Promote Pancreatic Ductal Adenocarcinoma Liver Metastasis

Dongqi Li [1,2†], Xiangyu Chu [3†✉], Fusheng Zhang [1†], Yongsu Ma [1], Weikang Liu [1], Ping Li [2,4], Xiaocui Fang [2,4], Chen Wang [2,4✉], Xiaodong Tian [1✉], Yanlian Yang [2,4✉], and Yinmo Yang [1✉]

[1]Department of Hepatobiliary ad Pancreatic Surgery, Peking University First Hospital, Beijing 100034, China

[2]CAS Key Laboratory of Standardization and Measurement for Nanotechnology, CAS Key Laboratory of Biological Effects of Nanomaterials and Nanosafety, National Center for Nanoscience and Technology, Beijing, China

[3]Department of General Surgery, Beijing Friendship Hospital, Capital Medical University, State Key Lab of Digestive Health, National Clinical Research Center for Digestive Diseases, Beijing, 100050, China

[4]University of Chinese Academy of Sciences, Beijing 100049, China

✉ Correspondence: Yinmo Yang: yangyinmosci@bjmu.edu.cn

Xiaodong Tian: tianxiaodong@pkufh.com

Yanlian Yang: yangyl@nanoctr.cn

Chen Wang: wangch@nanoctr.cn

Xiangyu Chu: chuxyyyyy96@163.com

† Dongqi Li, Xiangyu Chu, and Fusheng Zhang contributed equally to this work.

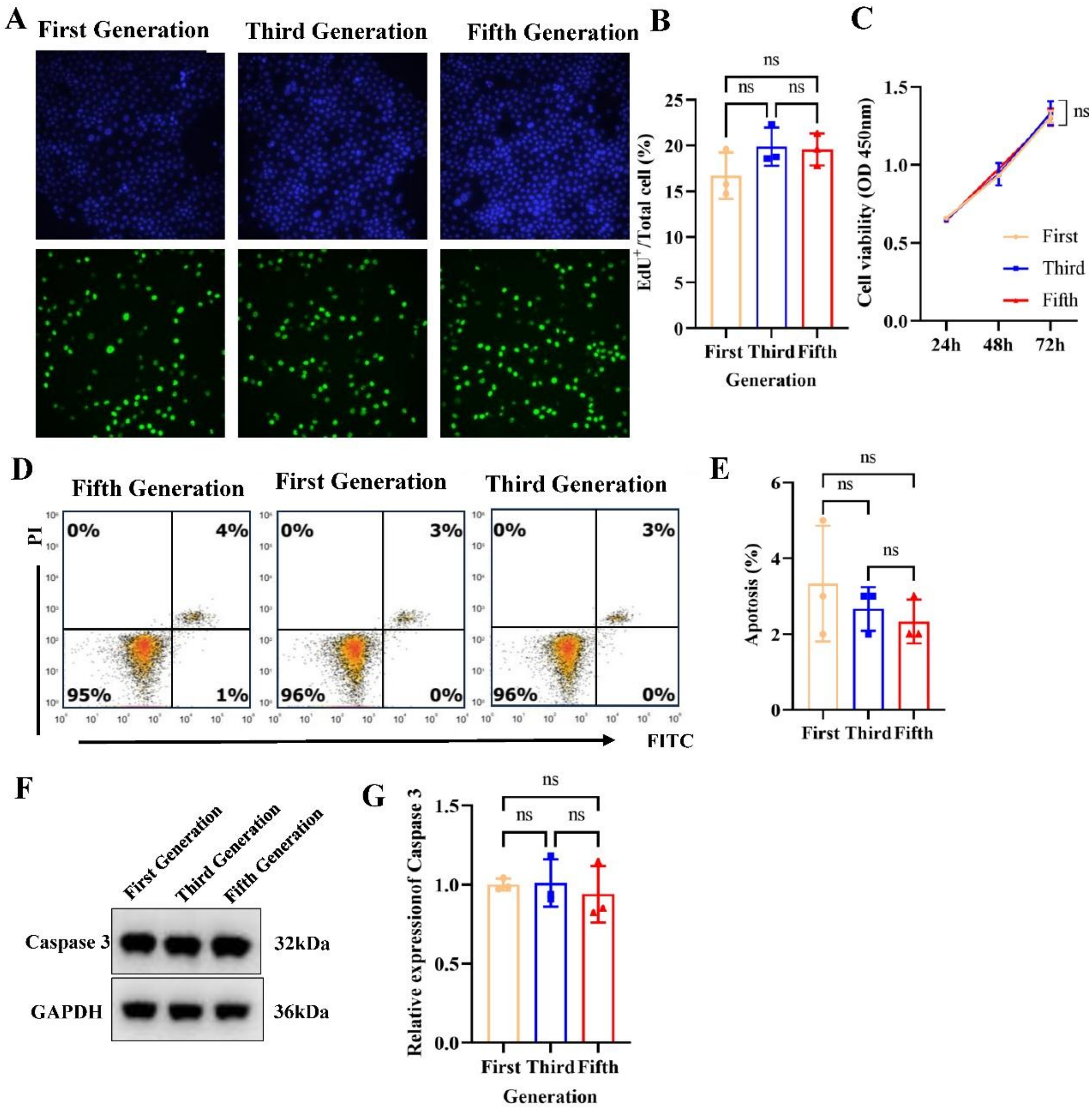

Fig. S1. **(A-B)** EdU staining and quantitative analysis of the first, third, and fifth passages of BxPC-3 (A) cells. Scale bar, 100 μm. **(C)** CCK8 analysis of the first, third, and fifth passages of BxPC-3 (A) cells. **(D-E)** Flow cytometry assay and quantitative analysis of the first, third, and fifth passages of BxPC-3 (A) cells. **(F-G)** Western blot analysis and quantitative analysis of caspase-3 in BxPC-3 (A) cells at the first, third, and fifth passages.

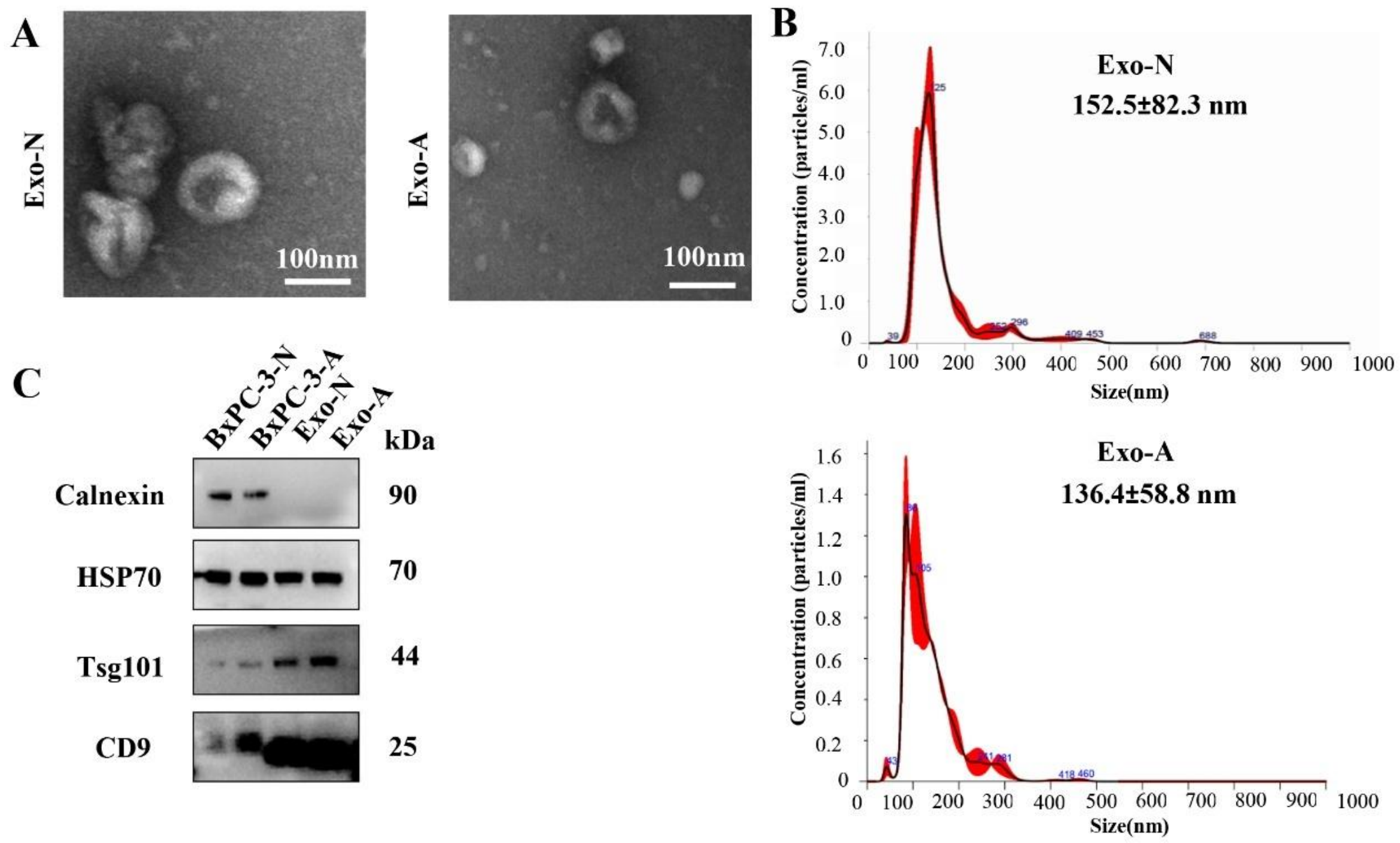

Fig. S2. Exo-A and Exo-N concentration and characterization. **(A)** TEM of Exo-A and Exo-N isolated from BxPC-3 (N/A) cells, scale bar = 100 nm. **(B)** Representative NTA images illustrating the size and particle distribution of Exo-A and Exo-N. **(C)** Isolated Exos exhibited expression of key exosomal proteins relative to parental cells. All experiments were performed with 3 independent replicates. $*p < 0.05$, $**p < 0.01$, $***p < 0.001$, $****p < 0.0001$.

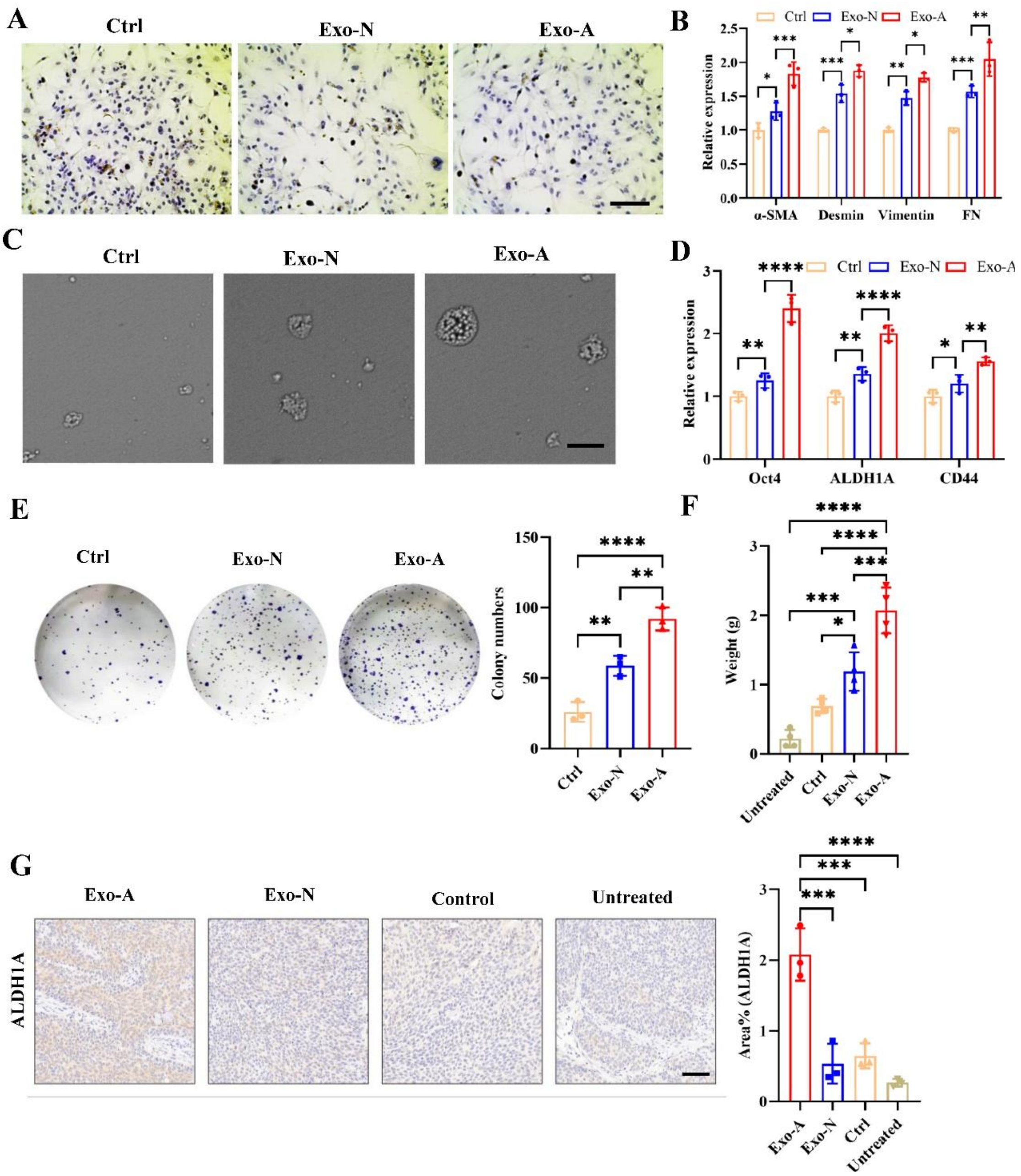

Fig. S3. Exo-A remodeled the ECM to promote PDAC stemness. **(A)** Representative images of oil red O staining after pre-treated with Exo-A and Exo-N, scale bar, 100 μm. **(B)** Quantitative analysis of α-SMA, Desmin, Vimentin and FN expression in LX-2 cells treated with Exo-A and Exo-N. **(C)** Quantitative analysis of tumor-sphere formation of PDAC cells cultured on ECM remodeled by LX-2 cells pretreated with Exo-A and Exo-N. Scale bar, 100 μm. **(D)** Quantitative analysis of CD44, ALDH1A and Oct4 expression in in PDAC cells co-cultured with remodeled ECM by LX-2 cells pretreated with Exo-A and Exo-N. **(E)** Representative images of colony formation assays for PDAC cells after ECM remodeling of Lx-2 cells. **(F)** Quantification of tumor weights. **(G)** IHC results and statistical analysis of ALDH1A of subcutaneous grafted tumors. Scale bar, 100 μm. $*p < 0.05$, $**p < 0.01$, $***p < 0.001$, $****p < 0.0001$. Abbreviations: PDAC,

pancreatic ductal adenocarcinoma; IHC, immunohistochemistry; ECM, extracellular matrix.

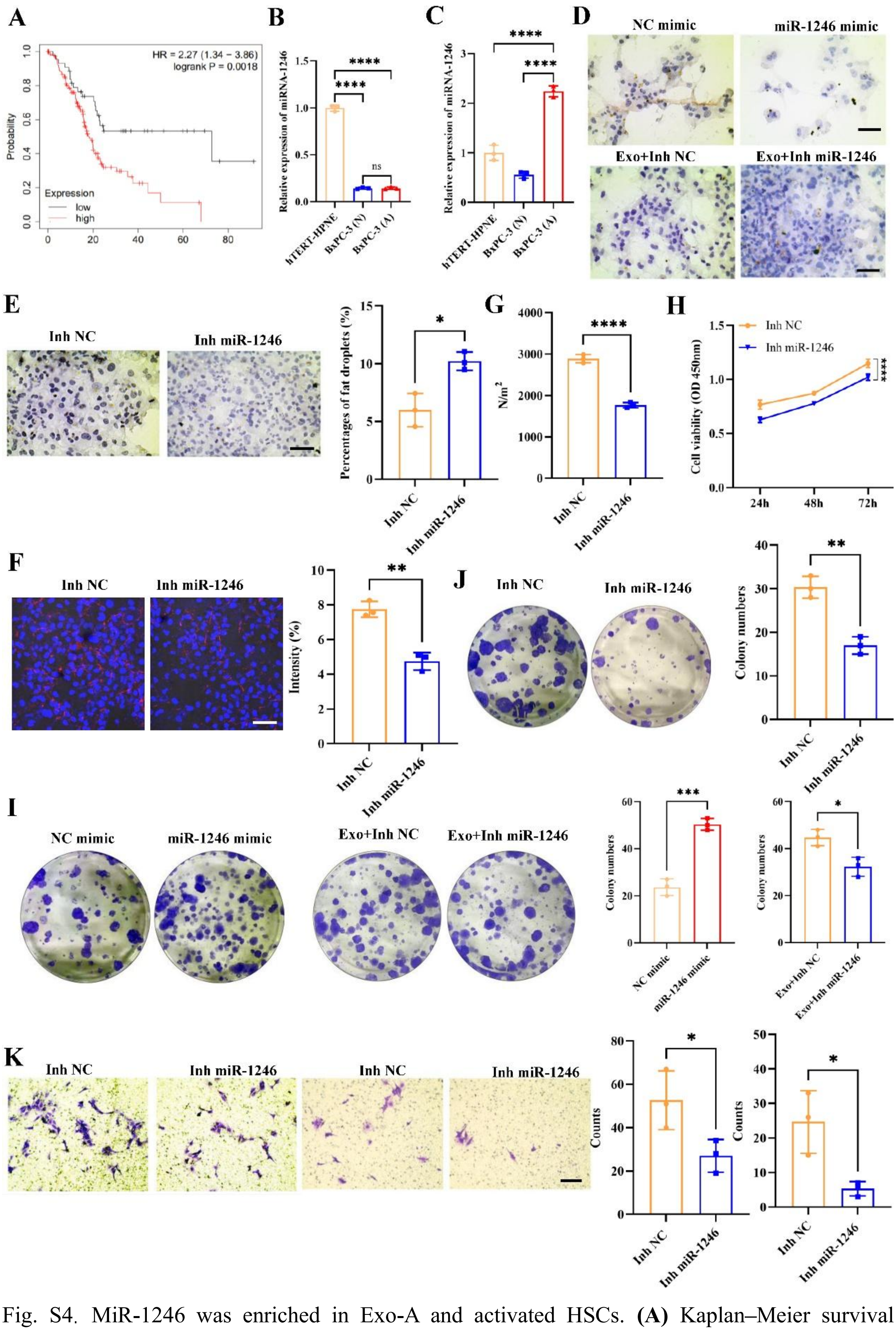

Fig. S4. MiR-1246 was enriched in Exo-A and activated HSCs. **(A)** Kaplan–Meier survival analysis comparing patient outcomes based on miR-1246 levels, with statistical significance

determined using the Log-rank test ($p < 0.01$). **(B-C)** The qRT-PCR analysis of cellular and exosomal miR-1246 expression in hTERT-HPNE, BxPC-3 cells under normal and acidic conditions, and relative Exos, with U6 as the endogenous control. **(D-E)** Representative images and statistical results of oil red O staining after overexpressing/silencing miR-1246, scale bar, 100 μm. **(F)** Representative images of IF analysis. The fluorescence intensity of Collagen I (red) was increased after transfecting miR-1246 inhibitors. Scale bar, 50 μm. **(G)** The statistical results of ECM stiffness of Lx-2 cells after treating them with miR-1246 inhibitors. **(H)** CCK-8 analyzed the proliferative capacity of Lx-2 cells after inhibiting miR-1246. **(I-J)** Representative images of colony formation assays showing LX-2 cell proliferation. **(K)** Transwell assays was performed to assay the capacity of migration and invasion of Lx-2 cells after silencing miR-1246, scale bar, 100 μm. $*p < 0.05$, $**p < 0.01$, $***p < 0.001$, $****p < 0.0001$. Abbreviations: WB, western blot; IF, immunofluorescence; ECM, extracellular matrix; FN, fibronectin; α-SMA, alpha-smooth muscle actin; CCK-8, cell counting Kit-8; HSC, hepatic stellate cell

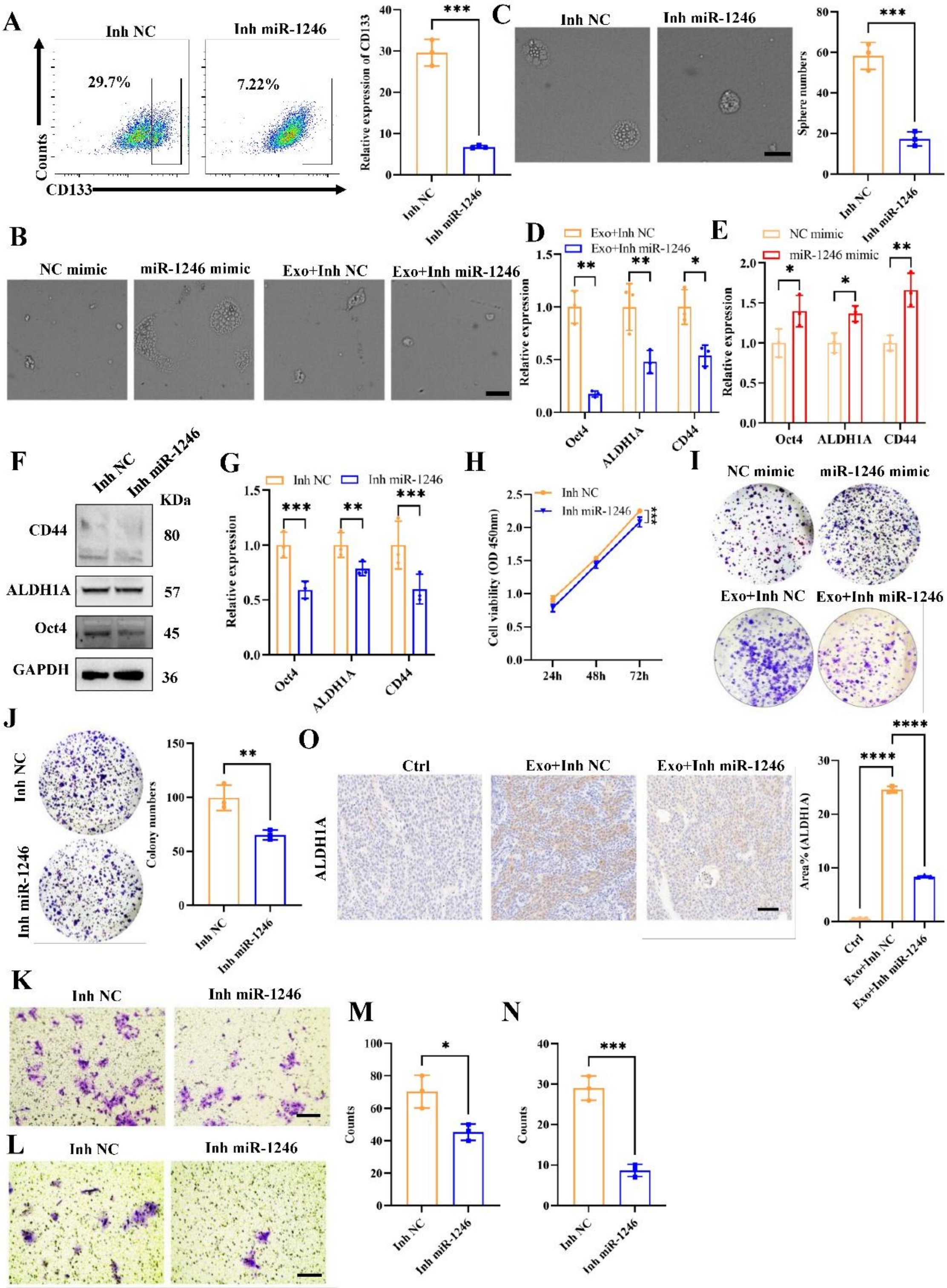

Fig. S5. MiR-1246 remodeled the ECM of HSCs to promote PDAC stemness. **(A)** Flow cytometry was performed to validate the ECM remodeling of Lx-2 cells after inhibiting miR-1246. **(B-C)** The representative images and statistical results of remodeled ECM of Lx-2 cells after overexpressing and silencing miR-1246 on the tumor-sphere formation of PDAC cells. Scale bar, 100 μm. **(D-G)** WB results and quantitative analysis revealed the expression of CD44, ALDH1A, Oct4 of PDAC cells after co-culture with remodeled ECM of Lx-2 cells transfected with miR-

1246 mimics and inhibitors. **(H)** CCK-8 analyzed the proliferative capacity of remodeled ECM on PDAC cells. **(I-J)** Representative images of colony formation assays for remodeled ECM on PDAC cells. **(K-N)** Transwell assays was performed to assay the capacity of migration and invasion of remodeled ECM on PDAC cells, scale bar, 100 μm. **(O)** IHC results and statistical analysis of ALDH1A of subcutaneous grafted tumors. Scale bar, 100 μm. $*p < 0.05$, $**p < 0.01$, $***p < 0.001$, $****p < 0.0001$. Abbreviations: CCK-8, cell counting Kit-8; HSC, hepatic stellate cell; PDAC, pancreatic ductal adenocarcinoma; WB, western blot; IHC, immunohistochemistry;

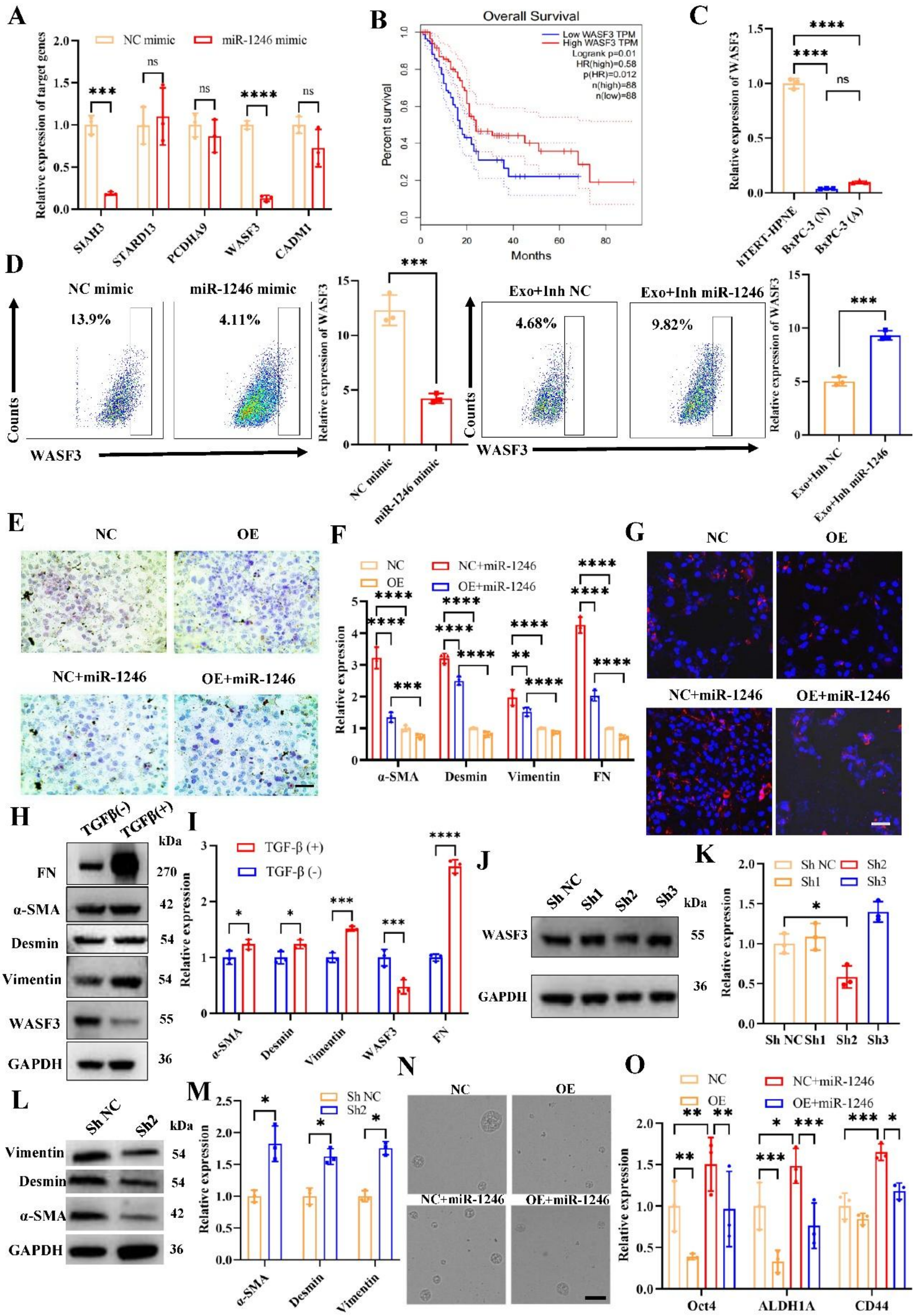

Fig. S6. MiR-1246 targeted WASF3 to promote HSCs activation and ECM remodeling. **(A)** The expression of selected target genes of miR-1246 in Lx-2 cells by qRT-PCR, with GAPDH as the endogenous control. **(B)** Kaplan-Meier survival curves comparing PDAC patient outcomes based on WASF3 levels, with statistical significance determined by the Log-rank test ($p < 0.01$). **(C)** The

expression of WASF3 in BxPC-3 (N/A) cells by qRT-PCR, with GAPDH as the endogenous control. **(D)** Flow cytometry was performed to validate the expression of WASF3 on Lx-2 cells after overexpressing miR-1246 and inhibiting miR-1246 pre-stimulated with Exo-A. **(E)** The representative images of oil red O staining following the transfection of miR-1246 in Lx-2 cells pre-treated with WASF3 NC/OE plasmids. **(F)** Quantitative analysis of α-SMA, Desmin, Vimentin and FN expression in LX-2 cells transfected with miR-1246 after pretreatment with WASF3 NC/OE plasmids. **(G)** Representative images of IF assays for Lx-2 cells after transfecting miR-1246, which were pre-treated with WASF3 NC/OE plasmids. Scale bar, 50 µm. **(H-I)** WB and Quantitative analysis of α-SMA, Desmin, Vimentin, WASF3 and FN expression in LX-2 cells treated with TGF-β. **(J-K)** WB and quantitative analysis of WASF3 in LX-2 cells transfected with WASF3 sh NC/sh1-3 plasmids. **(L-M)** WB and quantitative analysis of α-SMA, Desmin, Vimentin and FN expression in LX-2 cells transfected with miR-1246 after pretreatment with WASF3 sh NC/sh2 plasmids. **(N)** Representative images of remodeled ECM of Lx-2 cells overexpressed miR-1246 and silenced it pre-treated with Exo-A on the tumor-sphere formation of PDAC cells. scale bar, 100 µm. **(O)** Quantitative analysis of the expression of CD44, ALDH1A, and Oct4 in PDAC cells co-cultured with remodeled ECM from LX-2 cells pretreated with WASF3 NC/OE plasmids. $*p < 0.05$, $**p < 0.01$, $***p < 0.001$, $****p < 0.0001$. Abbreviations: WASF3, Wiskott–Aldrich syndrome protein family member 3; NC, negative control; OE, overexpression; qRT-PCR: Quantitative reverse transcriptase PCR

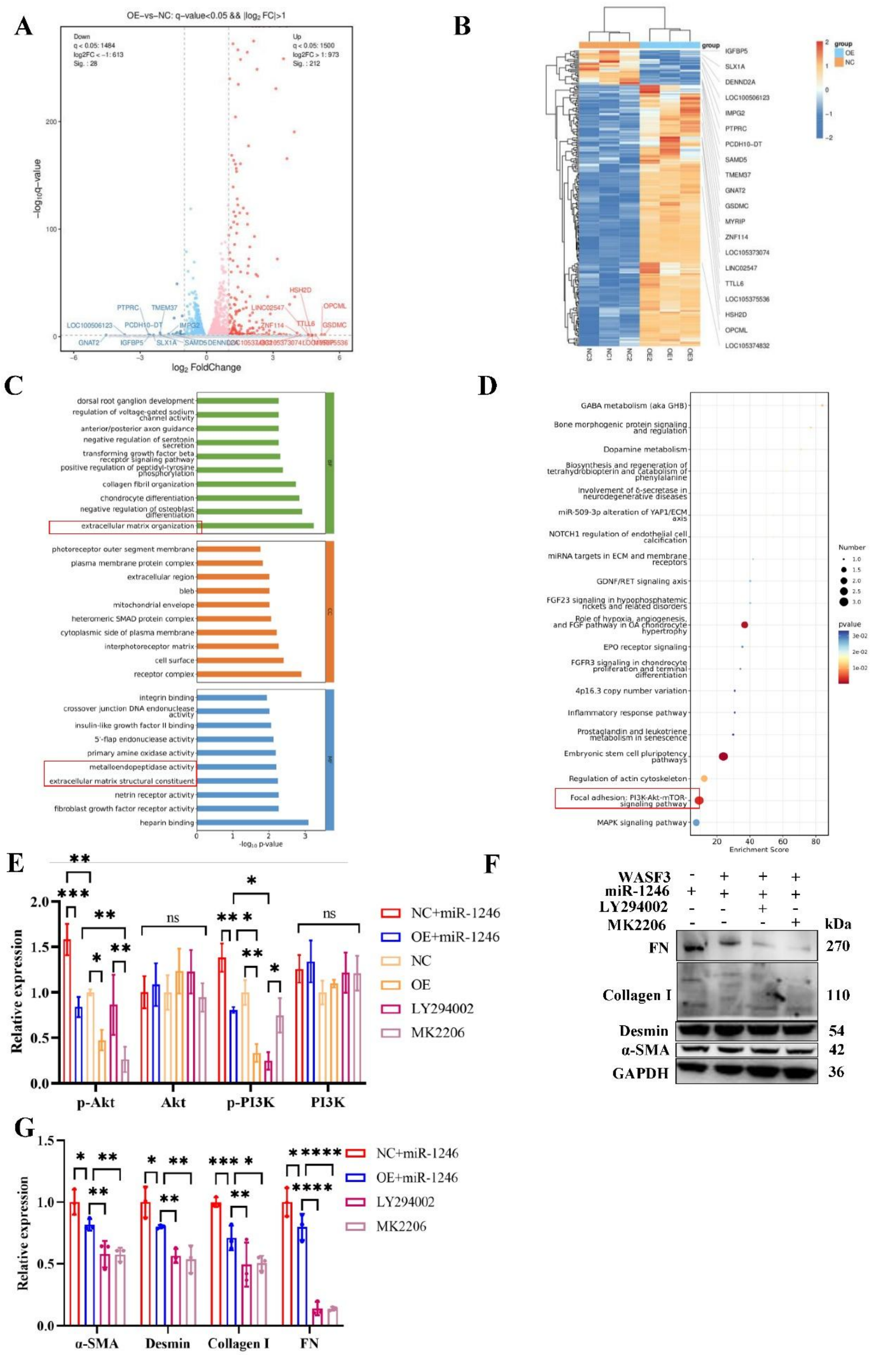

Fig. S7. TDEs miR-1246 targeted WASF3 to activated PI3K-Akt pathways to promote HSCs' activation and PDAC liver metastasis. **(A)** Volcano plot of selected differentially regulated genes in WASF3-overexpressing plasmids treated Lx-2 cells vs untreated Lx-2 cells. The vertical dashed

line showed an adjusted $p < 0.1$, and the horizontal dashed lines indicated an absolute log2 FC < 1. **(B)** The heat-map represents differential genes in WASF3-overexpressing group versus NC group. **(C)** GO term analysis identified significant functional enrichment among the differentially expressed genes in WASF3-OE cells versus NC controls. **(D)** The bubble plot represents pathway enrichment analysis performed on genes differentially downregulated in WASF3 OE group. **(E)** Quantitative analysis of protein expression in the PI3K/AKT signaling pathway in LX-2 cells transfected with miR-1246 and pretreated with WASF3 NC/OE plasmids (including PI3K inhibitor-LY294002, and Akt inhibitor-MK2206). (F-G) WB analysis and quantitative assessment of α-SMA, Desmin, and Collagen I expression in LX-2 cells transfected with miR-1246, following pretreatment with WASF3 NC/OE plasmids, as well as with the PI3K inhibitor LY294002 and the Akt inhibitor MK2206. Abbreviations: NC, negative control; OE, overexpression
